# Combined topological and spatial constraints are required to capture the structure of neural connectomes

**DOI:** 10.1162/netn_a_00428

**Published:** 2025-03-05

**Authors:** Anastasiya Salova, István A. Kovács

**Affiliations:** Department of Physics and Astronomy, Northwestern University, Evanston, IL, USA; NSF-Simons National Institute for Theory and Mathematics in Biology, Chicago, IL, USA; Northwestern Institute on Complex Systems, Northwestern University, Evanston, IL, USA; Department of Engineering Sciences and Applied Mathematics, Northwestern University, Evanston, IL, USA

**Keywords:** Maximum entropy, Distance dependence, Physical constraints, Neural connectome, Neural contactome, Graphlets

## Abstract

Volumetric brain reconstructions provide an unprecedented opportunity to gain insights into the complex connectivity patterns of neurons in an increasing number of organisms. Here, we model and quantify the complexity of the resulting neural connectomes in the fruit fly, mouse, and human and unveil a simple set of shared organizing principles across these organisms. To put the connectomes in a physical context, we also construct contactomes, the network of neurons in physical contact in each organism. With these, we establish that physical constraints—either given by pairwise distances or the contactome—play a crucial role in shaping the network structure. For example, neuron positions are highly optimal in terms of distance from their neighbors. Yet, spatial constraints alone cannot capture the network topology, including the broad degree distribution. Conversely, the degree sequence alone is insufficient to recover the spatial structure. We resolve this apparent mismatch by formulating scalable maximum entropy models, incorporating both types of constraints. The resulting generative models have predictive power beyond the input data, as they capture several additional biological and network characteristics, like synaptic weights and graphlet statistics.

## Author Summary

We investigate the interplay of the spatial and topological structure of millimeter-scale neural connectomes in fly, mouse, and human. As a spatial observation, we demonstrate that the probability of synaptic connection decays exponentially with distance. Additionally, we show that the wiring length in neural connectomes is highly optimal. To quantify the physical constraints on synapse formation, we construct the physical contact network for each organism and demonstrate that contact edge probability follows the same exponential functional form as the connectome. At the same time, we show that spatial constraints are necessary but not sufficient to reconstruct the connectome topology. We present maximum-entropy models capturing key spatial and topological aspects of the connectomes and demonstrate their predictive power beyond the input data.

## INTRODUCTION

Network representations of the brain offer a key to relating its multiscale structure to its dynamics and function (D. L. Barabási et al., 2023; Bassett & Sporns, 2017; Telesford, Simpson, Burdette, Hayasaka, & Laurienti, 2011). The widespread availability of the data at the scale of interregional and interareal connections (Chiang et al., 2011; Kötter, 2004; Lanciego & Wouterlood, 2011; Markov et al., 2014; Oh et al., 2014; Scannell, Blakemore, & Young, 1995; Zingg et al., 2014) made it possible to distill fundamental design principles of brain organization (Betzel, Griffa, Hagmann, & Mišić, 2019; Bullmore & Sporns, 2009, 2012; Horvát et al., 2016). Such macroscopic and mesoscopic networks must emerge from cellular-level synaptic networks—neural *connectomes*. Yet, up until recently, analyses of neural connectomes have been severely limited in their scope (Bassett et al., 2010; Chen, Hall, & Chklovskii, 2006; Gushchin & Tang, 2015; Maertens, Schöll, Ruiz, & Hövel, 2021; Y. Song, Zhou, & Li, 2021; Yan et al., 2017). The advent of volumetric brain reconstructions opens the possibility to map and model brain networks in a bottom-up manner (Haber, Wanner, Friedrich, & Schneidman, 2023; Lynn, Holmes, & Palmer, 2024b). Recent advances and major collaborative efforts in experimental, image analysis, and machine learning techniques have led to an unprecedented amount of nanometer-resolution brain datasets that span a variety of organisms (Dorkenwald et al., 2024; Helmstaedter, 2013; Hildebrand et al., 2017; Motta et al., 2019; Winding et al., 2023).

**Figure 1.**
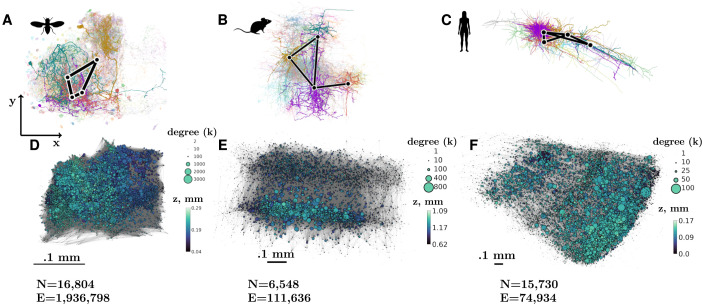
Visualization of the connectomes in the *xy* plane (axes shown on the left). A–C: subnetworks of fruit fly, mouse, and human neural connectomes. Neurons corresponding to network nodes (black dots) are shown in bright colors. A subset of neighbors of these neurons are shown in dimmer colors. D–F: full connectomes. Node positions correspond to the location of the “center of mesh” of individual neurons—the average position of their mesh vertices, see Synaptic Network Construction: Spatial Properties section. Node color and size correspond to undirected degree *k* and *z* position, respectively. Edges are shown in light gray. We also provide the number of nodes (*N*) and edges (*E*) in each network.

Here, we aim to quantify and compare the neural connectomes across organisms and understand the common design principles that lead to their complex structure. We focus on the roughly millimeter-scale connectomes of the adult fly, mouse, and human brains (The MICrONS Consortium et al., 2023; Scheffer et al., 2020; Shapson-Coe et al., 2024). These datasets allow us to compare the microscopic structure of the human brain to mammalian and even nonmammalian brains (Van den Heuvel, Bullmore, & Sporns, 2016). As an invertebrate model, we use the fruit fly hemibrain dataset, capturing most of the central brain of the adult female *Drosophila melanogaster* (Scheffer et al., 2020). As a mammalian model, we use the male mouse brain dataset that spans multiple cortical visual areas (The MICrONS Consortium et al., 2023). Finally, we use the reconstruction of a sample from the temporal lobe of the cerebral cortex in the female human brain (Shapson-Coe et al., 2024) to analyze the microscopic structure of the human connectome. We consider the undirected unweighted versions of the connectome obtained from these three datasets, where each edge represents the presence of some chemical synapses. The basic properties of these networks, each containing thousands of nodes and up to millions of edges (see Figure 1), are summarized in Table 1. The fruit fly neural connectome comprises 13% of the approximately 120,000 fruit fly neurons (Dorkenwald et al., 2024). In comparison, the mouse and human connectomes contain much smaller fractions of roughly 100 billion neurons in the human brain (Azevedo et al., 2009) and 100 million neurons in the mouse brain (Herculano-Houzel, Mota, & Lent, 2006).

**Table 1.** Properties of the synaptic and contact networks

	**Soma size**	**Contact thr.**	**# nodes**	**Network type**	**# connected nodes**	**# nodes in LCC**	**# edges**	**Density, %**	**Max. degree**	**Mean degree**	**Median degree**	**Wiring len. ratio**
fly	2.47 *μ*m	40 nm	16,804	synaptic contact	16,804	16,804	1,936,798	0.34	3,097	230.52	195	0.24
					16,804	16,804	7,747,751	1.37	7,335	922.13	806	
mouse	5.34 *μ*m	41 nm	6,548	synaptic contact	6,261	6,224	111,636	0.13	881	34.10	23	0.07
					6,507	6,485	1,373,875	1.60	2,042	419.63	418	
human	7.6 *μ*m	46 nm	15,730	synaptic contact	13,579	13,352	74,934	0.0032	138	9.53	6	0.03
					15,129	15,001	2,049,143	0.41	1,221	260.54	188.5	

To start, connectomes are inherently spatial (Barthélemy, 2011; Bassett & Stiso, 2018; Chklovskii, Schikorski, & Stevens, 2002; Horvát et al., 2016; Markov et al., 2011, 2013). Their structure is expected to be shaped by a balance of minimizing the wiring cost while maintaining complex topology, at least at large enough scales (Bullmore & Sporns, 2012; Ercsey-Ravasz et al., 2013; Horvát et al., 2016; Karbowski, 2001). In addition, neurons are complex fractal-like objects (Ansell & Kovács, 2024; Smith et al., 2021) embedded in three-dimensional space. Thus, they form complex *physical* networks (Pete, Timár, Stefánsson, Bonamassa, & Pósfai, 2024; Pósfai et al., 2024). To test the impact of physical contact on the connectome, we construct the neural *contactome* of each dataset (Braitenberg & Schüz, 1991; Kovács, Barabási, & Barabási, 2020; Peters & Feldman, 1976; Rees, Moradi, & Ascoli, 2017; Reimann, King, Muller, Ramaswamy, & Markram, 2015; Stepanyants & Chklovskii, 2005). There are several key open questions to address at the level of the neural connectome and contactome, such as: (a) Are the neural connectomes complex networks in the usual sense? For example, is their degree sequence heavy-tailed (see Degree Distribution and Distance Dependence section)? (b) To what extent is the connectome dictated by the spatial embedding? Specifically, how does the probability of forming connections change with the distance between neurons, and does this explain the neural connectome topology (discussed in the Connectome Models Incorporating Distance Dependence and Degree Sequence section and Degree Distribution and Distance Dependence section)? What aspects of the connectome structure are directly enforced by the contactome constraints (see Modeling the Connectome With Contact Constraints setion)? (c) Do neural connectomes exhibit optimal wiring, given the neuron positions and connectome topology (analyzed in the Degree Distribution and Distance Dependence section)?

As a final question, we ask, (d) Can we design simple generative network models that capture the main topological and spatial features of neural connectomes? Our analyses presented in the Connectome Models Incorporating Distance Dependence and Degree Sequence section and the Modeling the Connectome With Contact Constraints section suggest that a synergistic combination of spatial (e.g., contactome or distance dependence) and topological (e.g., degree sequence) constraints is required to form realistic models of neural connectomes. To incorporate these two types of constraints, we develop a range of scalable generative models using canonical maximum entropy network ensembles (Bianconi, 2021; Dichio & Fallani, 2023; Park & Newman, 2004; Robins, Pattison, Kalish, & Lusher, 2007; see Table 2). The combination of the intrinsically probabilistic nature of maximum entropy models and their ability to preserve local and global constraints makes them versatile for representing neural connectomes. Conceptually, such a framework is capable of capturing stereotypic brain connections together with individual variability in connectome datasets (Hiesinger & Hassan, 2018; Schlegel et al., 2024; Witvliet et al., 2021). In addition to preserving average quantities—soft constraints—we utilize the ability of maximum entropy models to respect hard constraints (Hao & Kovács, 2024; Kovács et al., 2020) by considering a class of models that only allow the formation of synapses between neurons in physical contact (see the Modeling the Connectome With Contact Constraints and Degree Preserving Model With and Without The Contact Constraint sections.

**Table 2.** List of maximum entropy models we use throughout the paper. Note that we only consider the maximum entropy models that, on average, preserve the total number of edges in the network

**Model name**	**Soft constraints**	**Hard contact constraint**
d	binned edge probability as a function of distance (*p*(*d*))	no
k	degree sequence (*k*_1_, …, *k*_*N*_)	no
k + L	degree sequence and total edge length (*k*_1_, …, *k*_*N*_, *L*)	no
c	–	yes
d + c	binned edge probability as a function of distance (*p*(*d*))	yes
k + c	degree sequence (*k*_1_, …, *k*_*N*_)	yes

**Figure 2.**
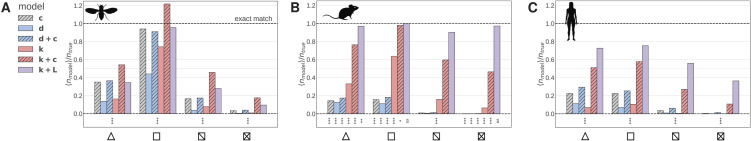
Comparing the graphlet counts in empirical connectomes and models based on 100 model realizations. Bar plots show the inverse fold changes (<*n*_*model*_>/*n*_*true*_) for the counts of the number of undirected triangle and square graphlets. The labels at the bottom correspond to the two-sided *p* values obtained from *z*-scores. *n.s.* (not statistically significant) corresponds to *p* > 0.05. *, **, *** correspond to *p* < 0.05, .01, and 0.001, respectively.

Models that aim to accurately represent the connectome need to capture its structure beyond the built-in network constraints. We show that the maximum entropy models that preserve distance and degree-based network features match other network properties, such as clustering, graphlet counts, and measures related to the length of shortest paths (see Figure 2 and Supporting Information Figure S1. Interestingly, our maximum entropy models have predictive power beyond what is dictated by the input data, as illustrated by the correlation between the edge probabilities in the models and the synaptic weights (see Supporting Information Figure S2). Additionally, models that include contact constraints capture the heterogeneity in distance dependence associated with neuron alignment in the mouse and human cortex, as shown in Supporting Information Figure S3. Altogether, we demonstrate the ability of simple network-based models to faithfully represent the connectomes across species, even in the absence of detailed organism-specific biological information.

## RESULTS

### Degree Distribution and Distance Dependence

**Figure 3.**
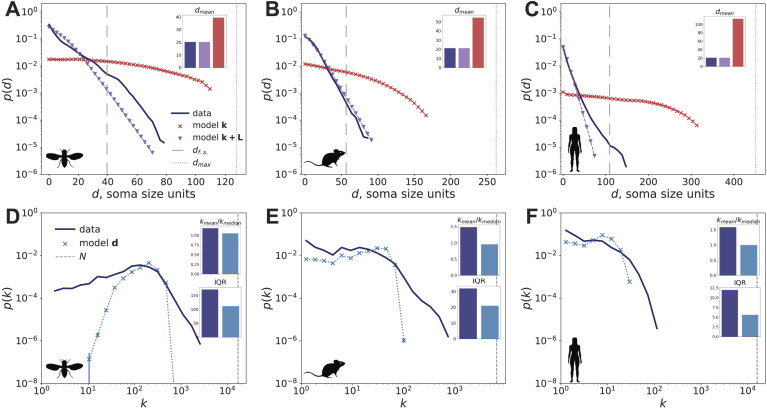
Distance and degree distribution of the connectome data and models. For a definition of soma size units used in the *d* axis, see Methods section. Each model distribution shows the average between 100 realizations, and the error bars represent standard deviations. A–C: distance distribution of the synaptic network data (dark blue), model k (red) and model k + L (purple). The vertical line at *d*_max_ corresponds to the largest distance between the neurons in the dataset. The vertical line at *d*_f.s._ corresponds to the distance between neurons above which the finite size of the experimental volume affects the distance dependence. This distance corresponds to the peaks of the pairwise neuron distance distributions shown in gray in Supporting Information Figure S9. A–C insets: mean interneuron distance between the connected pairs in data (dark blue) and models. D–F: degree distribution of the synaptic network data (dark blue) and model d (light blue). D–F, upper insets: ratio of mean degree to median degree in data and model d. D–F, lower insets: IQR of the degree distribution of data and model d.

Broad degree distributions are often hallmarks of complex networks, including biological networks (Amaral, Scala, Barthelemy, & Stanley, 2000; A.-L. Barabási & Albert, 1999; Lynn, Holmes, & Palmer, 2024a; Lynn et al., 2024b). As a first observation about the neural connectome topology, we note that the degree distributions of these networks, shown in Figure 3D–F, are broad but not scale-free. This contrasts with the distributions of edge weights—here, defined by the number of synapses between each pair of neurons—that are heavy-tailed consistently with the findings of Lynn et al. (2024b), as shown in Supporting Information Figure S4A–C. We also show that given the topology, the total wiring length—as quantified by the sum of Euclidean distances between all the pairs of connected neurons—is highly optimal compared with versions with randomly shuffled node positions (see Supporting Information Figure S5).

To characterize the spatial organization of connectomes, we model each neuron as a node whose position corresponds to the “center” of that neuron, given by the “center of mesh” of its surface, see the Synaptic Network Construction: Spatial Properties section. We then use the Euclidean distance between the neuron positions to represent the relative locations of pairs of neurons. As our first result, we find that the probability that a connectome edge exists between a given pair of neurons decays rapidly as a function of distance between these neurons. This is in line with the findings of exponential distance dependence in the *C. elegans* connectome (Arnatkeviciūtė, Fulcher, Pocock, & Fornito, 2018), mouse neural cell cultures (Yin et al., 2020), and interareal level connectomes in mammalian brains (Fornito, Arnatkevičiūtė, & Fulcher, 2019; Fulcher & Fornito, 2016; see also Distance Dependence and Model d section).

Qualitatively, the decay appears exponential, as demonstrated in the top row of Figure 3. Quantitatively, assuming exponential decay of a form *p*(*d*) ∝ *e*^−*d*/*d*_0_^, we estimate the constant *d*_0_ for the mouse connectome to be ∼0.05 mm. This scale differs from the scale of the exponential decay in interareal connectivity by an order of magnitude—*d*_0_ is estimated to be 2.179 mm for the mouse in Fulcher and Fornito (2016), which is larger than the linear dimensions of the mouse dataset studied here. To compare the characteristic distances *d*_0_ of the three organisms, we use the soma size as a distance scale. Estimating the soma sizes is discussed in Soma size estimation, and their numerical values are presented in Properties of the synaptic and contact networks. We estimate *d*_0_ to be 10, 10, and 12 in terms of some size units for fly, mouse, and human. Therefore, soma sizes set a distance unit that brings the characteristic network distances to the same scale.

Exponential decay is in line with the expectation that establishing and maintaining the synapses in neural connectomes is associated with wiring cost (Ahn, Jeong, & Kim, 2006; Bullmore & Sporns, 2012; Chen et al., 2006), and the wiring cost in empirical connectomes is lower than that of other networks with the same node positions and topology (Ercsey-Ravasz et al., 2013; Horvát et al., 2016). The simplest way to define the total wiring cost of the neural connectome is by summing up the Euclidean distances between the pairs of connected neurons. To assess the optimality of neuron placement without disrupting the network structure, we randomly shuffle the node positions while keeping the network topology fully intact (Ercsey-Ravasz et al., 2013; Horvát et al., 2016). The wiring length obtained from this randomization of the mouse connectome is about 3 times larger than the true wiring length (*p* < 0.001, see Supporting Information Figure S5 for the distribution of wiring lengths over 200,000 reshuffled samples). Similarly, the wiring length of the shuffled networks is about 2 times the true wiring length in the fly and more than 6 times the true wiring length in the human (*p* < 0.001), as illustrated in Supporting Information Figure S5. Therefore, we conclude that the observed wiring length is much shorter than expected by chance, given the connectome topology.

### Connectome Models Incorporating Distance Dependence and Degree Sequence

Distance dependence, degree sequence, and total wiring length can serve as building blocks (soft constraints) of simple generative models of the neural connectome (see first three models of Table 2). Here, we assess how well such generative models capture the spatial and topological structure of the connectome.

We find that the connectome degree sequence alone (model k, sometimes referred to as soft configuration model, shown in red in Figure 3 and discussed in the Degree Preserving Model With and Without The Contact Constraint section) does not imply the observed distance dependence. For instance, in model k, the average distance between the neighboring mouse neurons is 2.6 times larger than that of the mouse connectome. This means that, unlike the empirical connectome, model k is not as optimal in terms of the wiring cost—an observation that also holds for fly and human.

Next, we demonstrate that distance dependence alone is insufficient to predict the connectome structure. For instance, the observed degree distribution (Figure 3D–F, dark blue) is much broader than that coming from a distance-based model (model d) for all three organisms (Figure 3D–F, light blue). As an illustration, the interquartile range (IQR) of the degree distribution in the mouse connectome is significantly larger than the IQR in model d, 32.0 versus 20.9 (*p* < 0.001). Additionally, the mean to median degree ratio is 1.48 in the mouse connectome, compared with the much lower value of 0.95 in model d (*p* < 0.001). This indicates that the size of *hubs*—network nodes whose degree greatly exceeds the average—in the data is not fully captured by model d. For example, the number of partners of the highest degree hub—881 in the mouse, which constitutes 13% of the nodes in the network—is about an order of magnitude larger than that expected based on model d. Similarly, the hub degrees (see Table 1 for hub sizes in empirical connectomes) are significantly underestimated by model d in fly and human, as shown in Figure 3D–F.

Altogether, our results indicate competing implications of the broad degree distribution and the exponential distance dependence. To construct a network model that resolves this tension, in model k + L, we preserve the total distance *L* between the pairs of connected neurons—the overall wiring cost—in addition to maintaining the degree sequence (Halu et al., 2014), as discussed in Degree and wiring length preserving randomization. Unlike model k, model k + L leads to distance dependence that is qualitatively similar to what we observe in data (see top row of Figure 3, purple line), especially for the mouse. Note that model k + L requires estimating a distance parameter $d_{0}^{k+L}$ from data. Notably, the values of $d_{0}^{k+L}$ are similar for fly, mouse, and human (see the Degree and Wiring Length Preserving Randomization section) when measured in soma size units, reinforces the finding that soma size sets a natural length scale for neural connectome models.

As the next step, we consider the extent to which models d, k, and k + L generalize beyond the network features explicitly built into them. For example, we consider the counts of different *graphlets*—small connected subgraphs—of the connectome in data and models (Milo et al., 2002; Pržulj, 2007). Graphlet counts represent the higher-order local network structure beyond degree distribution, thus providing a sensitive measure of similarity between models and data (Pržulj, 2007). In Figure 2, we illustrate the four small undirected graphlets we consider—triangle, square, square with a diagonal edge, and a four-node all-to-all connected graph. As triangle counts indicate if the neighbors of connected neurons tend to be connected, an abundance of triangles can hint at similarity-driven connectivity. Similarly, an overrepresentation of squares with no diagonals suggests complementarity-driven wiring rules (Kovács et al., 2019). In general, the graphlet counts are better matched by the k + L model (violet), than by models d and k (unshaded blue and red, respectively; Figure 2).

In addition to graphlet patterns, we consider other local and global network measures commonly used in network neuroscience (Rubinov & Sporns, 2010) that we summarize in the Network Measures section. Global measures such as the network diameter and average shortest path length are well-captured by model k + L across the board, with model d and even model k often closely capturing these properties as well, as demonstrated in Supporting Information Figure S1. However, the advantage of incorporating a combination of network topology and spatial structure in model k + L is most evident for local network measures such as local efficiency, transitivity, and clustering coefficient. For instance, the average local efficiency—a measure of how fault tolerant the system is with respect to individual node removal (Latora & Marchiori (2001)—is relatively low in models k and d, but matches the empirical connectome better in case of model k + L, demonstrating an especially good agreement for the mouse. In contrast, a global analog of this efficiency measure is slightly overestimated by models k and d.

So far, we discussed how well the connectome models reproduce the empirical network structure and spatial organization. Another relevant question is the extent to which the models d, k, and k + L capture the existence of individual synapses—a question relevant to network link prediction. In other words, what is the performance of our models in a binary classification task of predicting whether two neurons form a synapse? To address this question, we first present the receiver-operating characteristic (ROC) curve, which demonstrates the relationship between the true and false positive rates, in Supporting Information Figure S6A–C. Additionally, we provide the precision-recall curve for this classification task, as shown in Supporting Information Figure S6D–F. This curve is a useful tool to assess the performance of our models given the severe data imbalance—synapses are only present between a small fraction of the available pairs of neurons. A higher area under the curve (AUC) corresponds to better performance in the classification task for both the ROC and precision-recall curves. As demonstrated in Supporting Information Table S2, the best performance is achieved in model k + L across datasets. Similarly, the likelihood of the observed connectome—as calculated using the corresponding maximum entropy model parameters—is the highest for model k + L (see Supporting Information Table S3).

Models d, k, and k + L do not explicitly preserve the physical contact constraint. For the mouse, 18%, 51%, and 64% of edges created by models d, k, and k + L, respectively, are contained in the contactome. The edge overlap arising from these models is larger than expected from Erdős-Rényi model—for example, 6.4% for the mouse. Similarly, model k + L preserves the contact constraint to a larger extent than other models in mouse and human, but the average fraction of overlap varies across the contactomes—being 32% for the fly, and 53% for the human.

To further assess the accuracy of models d, k, and k + L in capturing the contactome edges, we consider how well these models perform on a binary classification task that determines whether two neurons are in physical contact. We demonstrate the ROC curves and the precision-recall curves for this edge classification task in Supporting Information Figure S7, with the AUC values provided in Supporting Information Table S2. Model k performs better than chance, but worse than models d and k + L. For the mouse dataset, models d and k + L demonstrate similar performance in retrieving the contactome edges—AUC-ROC is 0.95, and AUC is 0.62 for the precision-recall curve—while for human and fly, model k + L clearly performs best. As we show in the Modeling the Connectome With Contact Constraints section, the maximum entropy framework also allows us to explicitly include the contact constraint to enforce that the connectome is a subnetwork of the contactome.

So far, we have shown that maximum entropy models with minimal inputs generalize to the properties of binary networks that are not explicitly included in the model. In addition to that, our models are also able to capture the “edge weights”—as defined by the number of synapses between pairs of neurons—similarly to the models in Haber et al. (2023). This is evident from Spearman’s rank correlation coefficients between edge probabilities *p*_*ij*_ and edge weights *w*_*ij*_, shown in Supporting Information Figure S2A–C. Models d, k, and k + L show positive statistically significant (*p* < 0.001) correlation with the empirical edge weights across datasets. When restricted to *p*_*ij*_ among the neural connectome edges (Supporting Information Figure S2D–F), model *k* +*L* shows the highest correlation with edge weights in fly, mouse, and human among the three models.

### Modeling the Connectome With Contact Constraints

**Figure 4.**
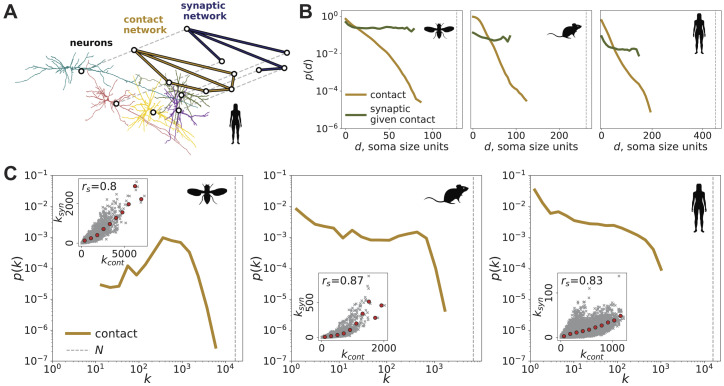
Relationship between the connectome and the contactome. A, from left to right: *xy* projection of mesh vertices of five human neurons and their center of mesh positions (black circles). Spatial contactome, or contact network, edges shown in dark yellow. Spatial connectome, or synaptic network, edges are shown in dark blue. The synaptic network and contact network have the same nodes, while synaptic edges are a subset of the contact network edges. The resulting network is naturally multilayer (multiplex) (Kivelä et al., 2014), as illustrated by connecting the representations of the same nodes with dashed lines. B: distance distribution of the contactome (dark yellow) and distance distribution of the connectome restricted to contactome edges (dark olive). Only the data points corresponding to at least 10 edges at a given distance bin are shown. C: degree distribution of the contactome. C, insets: degree of the node in the contactome (*x*-axis, *k*_cont_) versus its degree in the connectome (*y*-axis, *k*_syn_), shown in gray. Red circles represent the average values of *k*_syn_ corresponding to a binned range of *k*_cont_. We also provide the Spearman’s rank correlation coefficient *r*_*s*_ between the *k*_cont_ and *k*_syn_ variables.

The biological mechanisms required to form and maintain synapses might be different from those allowing neurons to be in physical contact. While synapses can be only observed between neurons in physical contact, physical contact alone might not dictate synapse formation, as parts of neurons could get in close proximity in passing. As a step toward decoupling physical contact from the effect of additional biological mechanisms, we construct neural contactomes from volumetric neuron reconstructions. First, we calculate the distances between pairs of neurons using their high-resolution mesh representation, as discussed in the Contact Network Construction section. Then, we use the distance threshold that recovers the edges of neural connectomes (see Supporting Information Figure S8) to construct contactomes. Thus, by construction, neural connectomes are subnetworks of the neural contactomes. The structure of a subset of the multilayer (multiplex network) human connectome-contactome network is illustrated in Figure 4A. To put that constraint in context, we note that the number of edges in the contactome *E*_cont_ is much larger than that in the connectome, *E*_syn_—specifically, *E*_cont_/*E*_syn_ ≈ 4, 12, and 27 for fly, mouse, and human. Still, contactome edges comprise less than 2% of all possible neuron pairs (see Table 1), thus significantly narrowing down the space of potential synaptic connections.

We find that the connectome and contactome exhibit similar exponential distance dependence (see Figure 4B and Supporting Information Table S1). Thus, distance dependence in neural connectomes appears to largely be a consequence of physical constraints. However, connectomes are not a random subset of contactomes, as indicated by the nonlinear relationship between the connectome and contactome degrees of individual neurons (see Figure 4 bottom row insets). Thus, the contactome constraints alone are insufficient to obtain accurate connectome models.

To further uncover the impact of physicality *alone* on the connectome in more detail (Pósfai et al., 2024), we consider random subnetworks of the contactome that preserve the number of edges in the connectome as a soft constraint (model c). This model does not explicitly preserve the distance dependence, wiring cost, or degree sequence. The average distance between neighboring neurons in model c is only 1.12 times larger than the true connectome wiring distance for the mouse. That indicates an improvement in capturing the wiring cost optimization compared to model k—another model that does not explicitly preserve distance dependence. The degree distribution of model c is still less broad (IQR of 29.0 for the mouse) and less dominated by hubs (mean to median degree ratio of 1.0 for the mouse) compared to the empirical connectome (IQR of 32.0, mean to median degree ratio of 1.48 for the mouse). This is an improvement compared to model d—another model that does not explicitly preserve the degree sequence. However, model c still underestimates the largest hubs.

The node degrees in the connectome and contactome (*k*_syn_ and *k*_cont_, *k*_syn_ ≤ *k*_cont_, shown in Figure 4C insets) are not independent. The Spearman’s rank correlation coefficient is large and positive for the three organisms: for example, its value is 0.87 for the mouse (*p* < 0.001), which shows that the neurons with high contactome degree *k*_cont_ tend to have high connectome degree *k*_syn_. However, the relationship between *k*_cont_ and *k*_syn_ appears to be superlinear (see the insets in Figure 4)—the nodes with many contact partners have a disproportionately large number of synaptic partners. Again, this demonstrates that the connectome is not a random subnetwork of the contactome, as in the random case, we would observe a linear relation.

**Figure 5.**
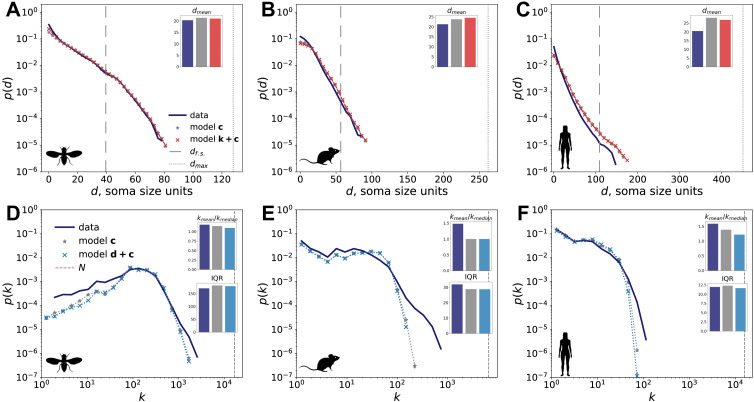
Distance and degree distribution of connectome data and models with contact constraints. Each model distribution shows the average between 100 realizations. A–C: distance distribution of the synaptic network data (dark blue), model c (gray) and model k + c (red). The vertical line at *d*_max_ corresponds to the largest distance between the neurons in the dataset. The vertical line at *d*_f.s._ corresponds to the distance between neurons above which the finite size of the experimental volume affects the distance dependence. This distance corresponds to the peaks of the pairwise neuron distance distributions shown in grey in Supporting Information Figure S9. A–C insets: mean interneuron distance between the connected pairs in data ans models. D–F: degree distribution of the synaptic network data (dark blue), model c (gray) and model d + c (light blue). D–F, upper insets: ratio of mean degree to median degree in data and model d. D–F, lower insets: IQR of the degree distribution of data and model d.

Yet, we find that the distance dependence in synaptic networks is largely captured by the contactome in all three organisms. As an illustration, the probability of a connectome edge given the contactome edge between a pair of neurons exists (olive line in Figure 4B) appears largely independent of distance. The quantitative agreement between the connectome and contactome distance dependence can be assessed by comparing the characteristic distance *d*_0_ assuming exponential decay *p*(*d*) =*αe*^−*d*/*d*_0_^—we estimate *d*_0_ to be 10 and 12 soma sizes in mouse connectome and contactome. A similar level of agreement in *d*_0_ estimates is observed in the fly and human (see the values provided in Supporting Information Table S1). To summarize, the distance dependence of connectomes follows the same trends as that of contactomes. As a result, model d + c—a model that preserves the distance dependence while respecting the contact constraint—does not perform better than model c in terms of capturing the degree distribution of the connectome (see Figure 5). Model d + c and model d have similar likelihoods of the empirical network (see Supporting Information Table S3), which is another indicator of distance dependence and contact constraints being highly redundant. Models c and d + c also underestimate the graphlet counts, as demonstrated in Figure 2.

Next, we introduce the model k + c that combines topological—in the form of degree sequence—and physical contact constraints. The average distance between the neighboring neurons in this model is 1.15 larger than that in the empirical mouse connectome. Thus, model k + c does not capture wiring cost constraints. The mismatch in wiring cost is significantly (*p* < 0.001) larger than that in model c—therefore, there is a trade-off between accurately capturing the wiring cost and preserving the degree sequence. However, model k + c improves on models k and c by better representing the higher-order connectome structure in fly, mouse, and human, as demonstrated by the graphlet counts in Figure 2. Similarly, model k + c is more accurate at reproducing other local connectome properties, such as local efficiency, transitivity, and clustering coefficient, as shown in Supporting Information Figure S1.

As an alternative way to assess the performance of models d + c and k + c, we determine whether they can predict which contactome edge is also present in the connectome. We again consider the ROC and precision-recall curves (see Supporting Information Figure S10), together with their AUC values (demonstrated in Supporting Information Table S2). We find that model k + c performs better in this classification task, as indicated by the AUC values. Model k + c also yields a higher likelihood of the empirical network than models c and d + c (see Supporting Information Table S3).

Similarly to models d, k, and k + L in the Connectome Models Incorporating Distance Dependence and Degree Sequence section, contact-based models partially capture the edge weights (number of synapses between pairs of neurons), even though no weight information is built into the models. Edge probabilities in model k + c show stronger Spearman’s rank correlation *r*_*s*_ with the number of synapses than the edge probabilities in d + c for all three organisms. For example, within the mouse connectome edges, *r*_*s*_ = 0.18 for model d + c and 0.36 for model k + c (*p* < 0.001 in both cases), as shown in Supporting Information Figure S2E. In general, models k + c and k + L are consistently the top models in capturing edge weights for the three organisms (see Supporting Information Figure S2A–F). Therefore, we conclude that among the considered models, models k + c and k + L are the best-performing models of the connectome across metrics. Both models reproduce the correct form of distance dependence, while exponential distance dependence is not explicitly built in.

However, distance dependence in neural connectomes is not fully homogeneous. For instance, the mouse and human connectomes we use come from cortical datasets, each containing several cortical layers (The MICrONS Consortium et al., 2023; Shapson-Coe et al., 2024), and the distance dependence within the layers can be different from distance dependence across the layers along the cortical columns. To treat the three datasets in a consistent way, we find the principal component vectors corresponding to the neuron alignment (see Supporting Information Table S1, Supporting Information Figure S3, and their captions). The details of distance dependence *p*(*d*_*i*′_), where *d*_*i*′_ is the distance along a specified component, differ across principal components (*i* = 1,2,3) (see Supporting Information Figure S3, panels B, E, H). Assuming *p*(*d*_*i*′_) ∝ *e*^−*d*_*i*′_/*d*_*i*_^, by construction it is true that *d*_1_ > *d*_2_ > *d*_3_. For example, we find *d*_1_ = 15, *d*_2_ = 10, and *d*_3_ = 8 in the mouse in soma size units (see Supporting Information Table S1). Likewise, we estimate the orientation-dependent distance scale in fly and human. In the human data, we find similar heterogeneity in *d*_1_ and *d*_2_—with a caveat that *d*_3_ is very small since the human brain sample is comparatively very thin. In the fly data—where, unlike in cortical layers of mouse and human, the neuron orientation is more radial, see Supporting Information Figure S11A—we also observe that distance dependence varies across the principal component directions. In the fruit fly, in contrast to other datasets, *d*_1_ appears to have a different functional form from *d*_2_ and *d*_3_.

Last, we compare the performance of the considered models on capturing the heterogeneity of distance dependence in empirical connectomes. As shown in Supporting Information Figure S3, panels C, F, and I, contactome (model *c*) qualitatively exhibits similar orientation dependence as the connectome, and the orientation dependence is also evident in model k + c. On the other hand, model k + L does not capture the heterogeneities in orientation dependence. Quantitatively, this is demonstrated in panels J–L of Supporting Information Figure S3. Thus, using the contactome constraint facilitates capturing the details of distance dependence, which are not captured even by model k + L that performs well based on other metrics.

## DISCUSSION

Connectomes are shaped by an interplay between spatial constraints and the need for complex topology that is essential to brain function (Bullmore & Sporns, 2012). To quantify the shared spatial properties of connectomes, we establish that distance dependence in undirected unweighted millimeter-scale neural connectomes is exponential. We also demonstrate that the characteristic distance scales are similar across species when expressed in soma-size units. Moreover, we show that the neuron positions are highly optimal in terms of minimizing the wiring cost. Driven by a biological hypothesis, we construct physical contact networks from volumetric data and show that exponential decay follows from the contactome structure. Similarly, the contactome captures the orientation dependence in the connectome. Thus, establishing accurate spatial or physical (Pete et al., 2024) generative models of the contactome can help elucidate the origin of the exponential distance dependence across species.

Recently, signatures of structural criticality—with critical exponents consistent across organisms—were observed in the three volumetric brain datasets we analyze here (Ansell & Kovács, 2024). Such universal properties of the brain anatomy are expected to have a profound impact on the spatial and topological organization of the contactome—and, consequently, connectome—and lead to similarities across species. Models of brain anatomy that belong to the same universality class also lead to models of the contactome, a direct input for some of our models. At this point, it remains an intriguing open question to determine whether structural criticality is required for the complex contactome structure presented here, and how it relates to brain function.

The contactomes we constructed for three organisms are useful beyond serving as spatial constraints for the connectome models. The contactomes can aid in predicting still missing chemical synapses in the reconstructions. In addition to chemical synapses, contactomes must contain electrical synapses—also known as gap junctions—that are much smaller and therefore hard to detect in raw volumetric data (Maeda et al., 2009). Thus, contactomes could serve as a useful constraint in gap junction prediction as well (Kovács et al., 2020). As detailed and accurate classification of neurites into axons and dendrites becomes routinely available in high-resolution volumetric datasets (Shapson-Coe et al., 2024), contactomes can become useful in analyzing the effects of subcellular wiring specificity on the connectome structure. Additionally, as directly comparing individual connectomes within the same species is already becoming feasible for complex organisms such as the fruit fly (Schlegel et al., 2024), the structure of the corresponding contactomes can be used as an extra measure of intraspecies variability. Last but not least, the novel approach of considering the multilayer connectome-contactome network goes beyond the standard spatial network framework and thus could ignite new research in network science.

As a first step in neural connectome topology analysis, we establish that the degree distribution is broad across datasets, yet not scale free. However, the degree distribution of the empirical connectomes does not follow from spatial constraints alone. Similarly, distance dependence is not implied by the connectome degree sequence. However, the models that resolve the mismatch by using a combination of spatial constraints and degree sequence—k + c and k + L—capture the connectome properties such as graphlet counts, efficiency, and distance dependence across species. These models demonstrate predictive power for connectome properties not explicitly built into them. For instance, the number of synapses—a proxy for edge weight—between the neurons is positively correlated with the edge probabilities implied by our models. The authors of Schlegel et al. (2024) demonstrate that in the fly brain, the connections with higher weight are significantly more likely to be found across hemispheres and across different organisms than edges with smaller weights—thus, more stereotyped edges are expected to be more likely to be captured by our maximum entropy models. Thus, our models have the potential to reliably capture the edges that are crucial to network function as well as the individual variability in lower-weight edges.

Note that the details of model performance vary across organisms—consistently with the findings of Haber et al. (2023). For instance, model k + L appears to be the best model to capture the basic structural features of mouse and human connectomes, while model k + c appears to demonstrate better performance on the fly data. The empirical distance dependence is not explicitly built into models k + L and k + c, but in both cases, it is well reproduced across organisms. However, model k + L does not capture the distance dependence heterogeneity that is picked up by model k + c. We conjecture that just like in the fruit fly, model k + c will perform better than model k + L once multiple brain regions are captured by the datasets. The discrepancies between models k + c and k + L and the empirical connectomes are evident from the statistically significant differences in their graphlet counts and network measures (see Figure 2 and Supporting Information Figure S1). These discrepancies can be informative of the wiring rules missing from our models, for example, wiring specificity based on neuron type, gene expression profiles, or the type of neurites in physical contact (Haber et al., 2023; Kovács et al., 2020; Udvary et al., 2022).

Exponential distance dependence arises naturally in models k + c and k + L. However, we use the empirical neural connectome degrees to obtain the appropriate neuron-level parameters that we then use in these generative models. At the same time, node degrees—and therefore the node-level model parameters—are nontrivially linked to structural properties of corresponding neurons (e.g., surface area, linear span, and their morphology in general). For instance, larger neurons are capable of forming synapses far away from their soma or center of mesh locations and, therefore, have more neighbors, while the location of positions of neighbors of small neurons is confined to a relatively smaller neighborhood. Linking the node-level parameters of generative models to observable structural and biological properties of neurons is a promising direction for future exploration that could link maximum entropy models to the bottom-up models of brain structure and development (Kaiser & Hilgetag, 2004; Oldham et al., 2022).

Our approach to analyzing volumetric brain data is in line with comparative connectomics (Van den Heuvel et al., 2016)—an emerging field that aims to uncover the general principles of brain network architecture and identify species-specific features of connectomes. The models we establish can serve as baselines to compare different neural connectome datasets. For instance, they can be useful in comparing different brain regions within the species, instances of the same brain region across healthy individuals, or differentiating the structure of the brain in healthy and diseased states.

Our analysis sets the stage for a thorough investigation of the existing and forthcoming high-quality volumetric brain reconstructions. The maximum entropy models we introduce are scalable, as they can be accurately solved using a simple iterative procedure (Vallarano et al., 2021). This property, as well as the ability to directly sample the models without using Markov-chain Monte Carlo sampling, makes applying our methodology feasible even for larger connectomes—for instance, the existing fruit fly brain reconstruction (Dorkenwald et al., 2024) or regions of the proposed complete reconstruction of the mouse brain (Abbott et al., 2020). In addition, maximum entropy models are flexible and can be in principle extended to include additional important features of connectome edges—for instance, directionality (Haber et al., 2023; S. Song, Sjöström, Reigl, Nelson, & Chklovskii, 2005), weight (e.g., the number of synapses between the neurons or synapse sizes) (Bianconi, 2009; Lynn et al., 2024b), sign (excitatory or inhibitory) (Gallo, Garlaschelli, Lambiotte, Saracco, & Squartini, 2024; Hao & Kovács, 2024), the type of neurites involved in synapse formation, or even multiway interactions between the neurons (Saracco, Petri, Lambiotte, & Squartini, 2022; Scheffer et al., 2020)—as well as their combinations. Incorporating these extra features as maximum entropy model constraints increases computational requirements, and incompleteness and biases in constraints may lead to biases in the maximum entropy models. However, analyzing the graphlet signatures of these more nuanced models provides an opportunity to gain additional insights into the structure of the connectome, as well as its relation to brain dynamics and function (Haber et al., 2023; Lizier et al., 2023). The graphlet analysis itself can also be generalized to explicitly include spatial information (Kim et al., 2012) in addition to topology, as well as node and edge labels, representing biologically relevant information. More detailed biological input could capture neuron gene expressions or cell types, or even different types of cell labels, such as neurons and glial cells (Fields et al., 2015), potentially incorporated into the maximum entropy models to reveal the effect of wiring specificity on connectome structure (Haber et al., 2023; Schneider-Mizell et al., 2023).

## METHODS

### Synaptic Network Construction: Topology

#### General considerations.

To construct a neuronal connectome, we need to define the nodes and edges of the network. In general, we want the nodes to correspond to individual neurons, while the edges should represent the presence of at least one synaptic connection. In practice, the fly, mouse, and human datasets we analyze contain reconstructed segments corresponding to parts of individual cells. Thus, each neuron can be present in more than one segment. To avoid representing a single neuron as multiple nodes, we restrict ourselves to segments that contain a soma in the experimental volume—this ensures each neuron is only counted once. Moreover, we only consider the cells with a *single* identified soma to avoid picking up cell merging errors. We present the basic properties of the connectomes we obtain in Table 1. Below, we discuss the specific steps we took to obtain the fly, mouse, and human neuron connectomes.

We use neuron meshes to define the positions (centers of mesh) of individual neurons, filter out the neurons cropped by the boundaries of the experimental volume, and define the contactome edges. The meshes of the fly, mouse, and human data sets we use are defined at different levels of detail (lod). Lod = 0 corresponds to the finest mesh, while lod = 3 corresponds to the coarsest representation of neuron surfaces.

#### Fly (see Scheffer et al., 2020).

We use the neurons that are labeled as “traced” and “uncropped” and whose soma positions within the experimental volume are available (16,804 neurons). The relevant part of the synaptic network is obtained from the compact connection matrix summary v1.2 release available at https://www.janelia.org/project-team/flyem/hemibrain. We use the version of the network with all of the detected synapses, which results in a total of 9,123,275 synapses in our connectome.

#### Mouse (see Elabbady et al., 2024; The MICrONS Consortium et al., 2023).

Note that the mouse data we used—including neuron ids, the meshes representing their volumetric structure, and synapse information—were obtained in September 2022. Since then, the datasets have been edited and improved, as discussed in The MICrONS Consortium et al. (2023). We start by obtaining soma information from the “nucleus_neuron_svm” table using CAVE, as discussed in https://github.com/AllenInstitute/MicronsBinder/blob/master/notebooks/mm3_intro/CAVEsetup.ipynb (Elabbady et al., 2024). We then filter the cells by having exactly one labeled soma. Finally, we only consider the cells labeled as “neuron.” There are 64,360 cells in the mouse dataset that satisfy these conditions. However, many neurons in the resulting connectome are cropped by the experimental volume boundaries, hindering our ability to assess their size and full spatial extent. To make the network similar to the fly dataset and avoid the effects of cropping the neurons on the spatial and topological properties of the connectome, we identify and use the uncropped neurons.

To find the uncropped neurons, we first define the volume boundary by finding the regions in space where no cells are detected for each slice in *z* dimension and finding the boundaries of each region using the MATLAB Image Processing Toolbox. Then, we thicken the boundary (the thickness of the boundary we use is larger than the largest distance between mesh vertices for lod = 1 to ensure all the boundary crossing neurons are filtered out) and remove any “holes” that appear due to misalignment of different imaged *z* slices. As our next step, we identify the meshes that do not have any vertices on the boundary at lod = 1. The resulting 9,118 cells become our uncropped neuron candidates.

Some of the segments among the uncropped neuron candidates do not appear to correspond to neurons. Specifically, some cells labeled as “neurons” in the dataset appear to be glial cells (e.g., see the purple cell in Supporting Information Figure S12, left panel). Other cells appear to only contain a fraction of the cell (e.g., see the blue object that looks like a soma in Supporting Information Figure S12, left panel) or arise from other segmentation errors. Plotting two spatial properties of neurons—their span and number of mesh vertices at lod = 3 (a proxy for their surface area)—reveals that the neuron candidates form clusters in this 2D space. To obtain the cluster boundaries numerically, we use the DBSCAN algorithm with parameters *ε* =.1 and min_samples = 30 (Pedregosa et al., 2011). This leads to five categories (shown in Supporting Information Figure S12)—two with relatively short span and relatively low number of mesh vertices (“small” and “incomplete” cells), one with a large number of mesh vertices and moderate cell span (“glia-like” cells), a large cluster containing 6,489 “neuron-like” cells, and unclassified cells that were not assigned to any of the clusters.

To validate our selection of the largest cluster as the one corresponding to neurons, we consider the 78 proofread neurons with extended axons and somas. While none of the proofread neurons are uncropped, their positions on the span versus number of mesh vertices plot (black triangles in Supporting Information Figure S12) overlaps with the location of the “neuron-like” cluster. Finally, we use the cells in “neuron-like” cluster together with 59 additional segments that were originally unclassified but are closest to the center of mass of the “neuron-like” cluster as the nodes of the connectome (6,548 nodes total). We obtain the synapses—163,188 in total—associated with these 6,548 neurons from https://bossdb-open-data.s3.amazonaws.com/iarpa_microns/minnie/minnie65/synapse_graph/synapses_pni_2.csv.

Furthermore, we assess the effect of only using the connections between the uncropped neurons on the connectome structure by considering our connectome degrees as a lower limit on node degrees. The “medium” and “upper” bound degree distributions are provided in Supporting Information Figure S13A—both of them are broad and nonpower law. The “intermediate” degree sequence—where we include all single-soma neuron neighbors of our fully contained neurons—shows high Pearson correlation with node degrees in our connectome (see Supporting Information Figure S13B).

#### Human (see Shapson-Coe et al., 2024).

We obtained the soma labels for individual neurons from gs://h01-release/data/20210601/c3/tables/somas.csv. For our analysis, we select the neurons that have a single labeled soma—15,730 cells in total. We acquired the list of synapses from the files in gs://h01-release/data/20210601/c3/synapses/exported/json. The synapse dataset also includes the labels of parts of the neuron involved in synapse formation (e.g., axon to dendrite). However, we do not perform any filtering based on this information and use the entire dataset containing 115,165 synapses.

The shape of human brain experimental volume is qualitatively different from that of the mouse or fly dataset. Namely, the size of the experimental volume in *z* dimension is much smaller than that in *x* and *y*. We can still identify the neurons that do not cross the experimental volume boundary (967 cells). Alternatively, we can also identify the neurons that do not cross the *xy* boundary while possible intersecting the boundaries of the experimental volume in *z* (7,176 cells). However, in both of cases, much of the network structure is lost (330 and 24,144 edges are preserved in a network with neurons fully contained within the *xy* boundary and the full experimental boundary, respectively) and the network is largely disconnected. Thus, we keep the entire human network in our analysis.

### Synaptic Network Construction: Spatial Properties

The connectome is inherently spatial. Individual neurons are complex objects embedded in physical space, whose spatial organization relative to other neurons affects who they can form synapses with. The simplest way to capture the neuron location is via its soma position, as illustrated by node positions in Supporting Information Figure S11. Then, the relative locations of neurons can be represented by the Euclidean distance between their somas. This connectome representation leads to the edge probability that clearly decreases with distance in mouse and human connectomes (see Supporting Information Figure S11 purple dash-dotted line, see the Soma Size Estimation for the definition of soma size units). However, the fly edge probability decreases very slowly at larger distances (approximately 20 to 120 soma sizes, as shown in Supporting Information Figure S11). This is largely the result of the distinct spatial organization of neurons in the fruit fly brain, where the somas (dots in Supporting Information Figure S11A and Figure S14) occupy the periphery of the brain, and the neurites are located closer to the center of the brain (Supporting Information Figure S11, see example neurons shown in green and purple) (Scheffer et al., 2020). Thus, the soma position in the fly is not representative of the neuron location as a whole.

To represent the neuron positions more effectively, we use the “center of mesh”—the center of mass of mesh vertices of individual neurons that we calculated at lod = 1 for the three organisms. This center of mesh can be thought of as the center of mass of the *surface* of individual neurons. Defining the distance between neurons as the Euclidean distance between the centers of mesh leads to a more rapid decay of the fly distance dependence and a narrower range of distances between the neurons in synaptic contact (Supporting Information Figure S11, dark blue dashed line). To a lesser extent, similar trends are seen in the mouse and human.

Note that the spatial connectomes we analyze are cropped—thus, distance dependence for *d* above a certain threshold does not represent the full neural connectome distance dependence at those distances *d*. To obtain a distance threshold, we consider the pairwise distances between all pairs of neurons for each organism (shown in gray in Supporting Information Figure S9). The peaks of these distance distributions roughly correspond to the distance threshold we seek—for example, we estimate it to be *d*_*thr*_ = 56 soma sizes for the mouse.

The spatial connectome we constructed can be used to estimate the distance scale *d*_0_ of the exponential decay *p*(*d*) ∝ *e*^−*d*/*d*_0_^ in the three organisms. Using the distance data below *d*_*thr*_, we estimate *d*_0_ = 10, 10, and 12 soma sizes for fly, mouse, and human respectively—the values are close across organisms. Without the distance threshold, these estimates become *d*_0_ = 9, 9, and 15 for fly, mouse, and human. We also expect the distance dependence to not be uniform—for example, distance dependence along the cortical columns could be different than distance dependence within the cortical layer in mouse and human datasets. This is quantified and illustrated in Supporting Information Table S1 and Supporting Information Figure S3.

Finally, we consider the effect of only including the edges between the uncropped neurons in the estimated mouse *d*_0_ value. To do so, we compare the distance dependence in the connectome with the distance dependence in a network that includes the edges between the uncropped neurons and cropped neurons with a soma in the volume, see Supporting Information Figure S13A inset. Here, we define distance as the Euclidean distance between somas. We get similar values for the two networks—*d*_0_ = 11 and 12 soma sizes.

### Soma Size Estimation

We define the soma size as a quantity roughly corresponding to the typical soma radius. To estimate the fly and mouse soma sizes from data, we collected the distances *d* from the center of the soma to physical contact locations. Using physical contact locations instead of synapse positions enables using more data points and ensures that the soma size is correctly estimated for the fruit fly, where no synapses are formed directly on the soma (Scheffer et al., 2020). We expect the typical soma size to be located at the peak of the distance distribution (shown as a histogram in gray in Supporting Information Figure S15).

The approach outlined above did not result in a clear peak in the human dataset (gray histogram, right panel in Supporting Information Figure S15). Fortunately, synaptic contacts in the human dataset are classified based on which parts of the pair of neurons (e.g., axon and soma) were involved in the formation of the synapse (Shapson-Coe et al., 2024). Once we restricted the data to only include the synapses connecting axons or dendrites of the presynaptic neuron to the soma of the postsynaptic neuron, a peak emerged.

To estimate the soma size *r*_soma_ from data, we perform kernel density estimation with linear kernel form and the bandwidth parameter of 0.2, 0.5, and 1.5 for fly, mouse, and human (dark blue line in Supporting Information Figure S15), and find the location of the peak of this function (*r*_soma_ ≈ 2.47*μ*m, 5.34*μ*m, and 7.6*μ*m for fly, mouse, and human respectively, shown in red in Supporting Information Figure S15) (Pedregosa et al., 2011). The fly soma size obtained using our methodology is in excellent agreement with the average of the soma radii provided in the janelia hemibrain dataset https://www.janelia.org/project-team/flyem/hemibrain for the neurons we consider (see the Synaptic Network Construction: Topology section): 2.467*μ*m.

Soma size units define a scale that unifies the three connectomes. For instance, the characteristic distance *d*_0_ obtained from the degree and wiring length-preserving maximum entropy model is approximately nine soma sizes across organisms (see the Degree and Wiring Length Preserving Randomization section), and the average distance between synaptically connected neurons is approximately 20 soma sizes (20.27 for fly, 21.19 for mouse, and 20.42 for human). Similarly, the scale of the distance dependence in both connectome and contactome—labeled as *d*_*tr*_ and $d_{tr}^{c}$ in Supporting Information Table S1—is of the same order of magnitude for the three organisms.

### Contact Network Construction

Establishing synapses between neurons relies on their surfaces being in close spatial proximity. This spatial constraint can be expressed by constructing physical contact networks—contactomes. We obtain the contactomes using the vertices of neuron meshes at lod = 1 for all three organisms. For each pair of neurons, we calculated the smallest pairwise distance *d*_*ij*,min_. Then, we applied the distance threshold *d*_*ij*,min_ < *d*_thr_ under which more than 99% of the synaptic edges are contained in the contact network, see the magenta line in Supporting Information Figure S8. Rounding up to the nearest nanometer, we get the thresholds of *d*_thr,fly_ = 40 nm, *d*_thr,mouse_ = 41 nm, and *d*_thr,human_ = 46 nm. All the edges representing pairs of neurons at a distance under this distance threshold are assigned to the contact network. Additionally, we add the 1% of the synaptic edges that are not currently contained in the contact network.

As expected from our procedure, the data-driven distance thresholds we obtained are in the order of magnitude of the size of the synaptic clefts in chemical synapses (Zuber, Nikonenko, Klauser, Muller, & Dubochet, 2005). These thresholds are much larger than the size of the intercellular space in gap junctions (Maeda et al., 2009). Thus, contactome edges contain more than one important mode of interneuronal communication. On the other hand, the thresholds we impose are significantly smaller than micrometer-level thresholds in Reimann et al. (2015)—there, the distance corresponds to the linear dimension of the dendritic spines. Even at the strict threshold values we imposed, most of the contactome edges do not correspond to the presence of a chemical synapse, as demonstrated by the network densities in Table 1. The connectome edges comprise 25%, 8%, and 0.8% of the contactome edges in fly, mouse, and human respectively.

The distance dependence in the contactomes is well approximated by an exponential. We estimate the distance scale *d*_0_, assuming *p*(*d*) ∝ *e*^−*d*/*d*_0_^, in the three organisms: *d*_0_ = 12, 12, and 15 soma sizes for fly, mouse, and human respectively. Remarkably, these distance values are similar to those estimated for the connectome (see the Synaptic Network Construction: Spatial Properties section and Supporting Information Table S1).

The contact networks we constructed are undirected and unweighted, but their construction can be generalized to account for these important structural aspects. For instance, weights could be assigned based on the area of the physical contact or the number of disconnected cell subregions in contact with each other for each pair of cells.

### Distance Dependence and Model *d*

Model d assigns the edge probability for a pair of nodes [*i*, *j*] based on the distance between them to match the empirical distance dependence *p*(*d*). Specifically, to approximate *p*(*d*), we bin the Euclidean distances between the neuron centers of mesh using 50 linear bins. For each bin *b* = [*d*_min_, *d*_max_], we calculate $p_{b}=\frac{N_{\text{edges},b}}{N_{\text{pairs},b}}$—the fraction of pairs of neurons at a distance within bin *b* that are connected by an edge in the empirical connectome. Then, for each pair of neurons [*i*, *j*] in the data set, we determine the distance bin *b* and form an edge with probability *p*_*b*_._._

Note that the definition of *p*(*d*) we use is different from the *p*(*d*) defined in some of the seminal spatial brain network literature (Ercsey-Ravasz et al., 2013; Horvát et al., 2016) formalizing the exponential distance rule (EDR) as a fundamental organizing principle in mammalian brain structure. In that body of work, the distance *d* is defined as a projection length along axon bundles—while in this manuscript, we consider the Euclidean distance between individual neurons’ centers of mesh (see gray and magenta lines in Supporting Information Figure S16). Additionally, they define *p*(*d*) as the distribution of axon length—in contrast, we consider the *connection probability* for neurons at a distance *d*, as discussed above. In our case, whether a pair of neurons is considered connected is defined by the presence or absence of synapses between them. In contrast, EDR does not explicitly account for interneuronal connections—instead, the number of axons between brain areas can indirectly account for the number of pairwise connections between corresponding neurons. EDR can be used to create weighted interareal connectomes. Similarly, the neuron-level data we use allows us to construct weighted neural connectomes. We demonstrate the weighted and unweighted distribution of Euclidean distances between pairs of connected neurons in Supporting Information Figure S16, magenta and teal lines. While the tail of these distributions looks similar to that of our distance dependence *p*(*d*), the behavior of these distributions is quantitatively different from *p*(*d*) at small distances *d*—namely, the region where the finite size of the experimental volume does not affect the inferred distance dependence (see Supporting Information Figure S9 and caption).

### Degree Preserving Model With and Without the Contact Constraint

The entropy of a network ensemble is defined as

$$S\left(G\right)=-\sum_{G}P\left(G\right)\ln P\left(G\right),$$ (1)

where the network ensemble assigns a probability *P*(*G*) to each network *G*. Maximum entropy network ensembles are a useful tool in network science. Inspired by information theory and statistical physics, they represent our partial knowledge of the network by preserving a set of constraints—for example, degree sequence—without making any assumptions about any other aspects of its structure.

As synaptic network data may be noisy, incomplete, and variable in time due to synaptic plasticity, we capture its structural properties by using the *canonical ensemble* that preserves network constraints on average. For instance, let soft constraints be the degree sequence $k_{1}^{*},\ldots ,k_{N}^{*}$ (model k, referred to as *soft configuration model* in literature). Then,

$$\langle k_{i}\rangle =\sum_{j}A{\left(G\right)}_{ij}P\left(G\right)=k_{i}^{*},$$ (2)

where *A*(*G*)*_ij_* stands for the elements of the adjacency matrix *A* of a network *G*. Another constraint comes from the fact that probabilities *P*(*G*) should add up to 1. Maximizing the entropy in Equation (1) with these constraints using the method of Lagrange multipliers (Park & Newman, 2004), we arrive at

$$P\left(G\right)=\frac{e^{-H\left(G\right)}}{Z},$$ (3)

where $H\left(G\right)\equiv \sum_{i}\theta_{i}k_{i}\left(G\right)=\sum_{i<j}\left(\theta_{i}+\theta_{j}\right)A{\left(G\right)}_{ij}$ is the Hamiltonian, and $Z\equiv \sum_{G}e^{-H\left(G\right)}=\prod_{i<j}\left(1+e^{-\theta_{i}-\theta_{j}}\right)$ is the partition function. From Equation (2), the explicit expressions for the Lagrange multipliers for the maximum entropy model can be obtained from $k_{i}^{*}=-\frac{1}{Z}\frac{\partial Z}{\partial \theta_{i}}$. Explicitly,

$$k_{i}^{*}=\sum_{j}p_{ij}=\sum_{j}\frac{e^{-\theta_{i}-\theta_{j}}}{1+e^{-\theta_{i}-\theta_{j}}},$$ (4)

where *p*_*ij*_ is the probability of *i* and *j* forming an edge. Note that these edge probabilities are independent of each other—thus, the network ensemble can be sampled by forming each edge *ij* with probability *p*_*ij*_. The parameters *θ_i_* can be calculated using a simple and efficient iterative scheme with an update rule

$$x_{i}^{t+1}=\frac{1}{k_{i}^{*}}\sum_{j}\frac{1}{1/x_{i}^{t}+x_{j}^{t}},$$ (5)

where *x*_*i*_ ≡ *e^θ_i_^*, for $k_{i}^{*}>0$ (Vallarano et al., 2021). If $k_{i}^{*}=0$, *p*_*ij*_ = 0 in this ensemble.

Contact constraints can be explicitly incorporated into the maximum entropy framework as hard constraints (model k + c): no edges (synapses) can exist between the nodes (neurons) that are not in physical contact. There, we explicitly take the hard contact constraint into account by forcing *p*_*ij*_ to be zero where no edge exists in the contact network. Then, the partition function is $Z=\prod_{i<j,i∼j}\left(1+e^{-\theta_{i}-\theta_{j}}\right)$, where *i* ∼ *j* indicates the existence of an edge in the contactome (Kovács et al., 2020). The Lagrange multipliers *θ_i_* and edge probabilities *p*_*ij*_ between the nodes in physical contact can then be obtained in the same way as for model k.

### Degree and Wiring Length Preserving Randomization

In spatial networks such as the brain, forming and maintaining edges is often associated with a wiring cost (Bullmore & Sporns, 2012). Possibly the simplest cost function is the linear function *f*(*d*_*ij*_) =*d*_*ij*_, where *d*_*ij*_ is the distance between the nodes. Following Halu et al. (2014), in our model k + L we impose soft constraints on the total wiring cost *L* in addition to preserving the degree distribution Equation (2):

$$\langle L\rangle =\sum_{i<j}A{\left(G\right)}_{ij}d_{ij}P\left(G\right)=L^{*}.$$ (6)

Maximizing the entropy of the network ensemble (Equation (1)) with constraints (Equation (6) and Equation (2)) using the method of Lagrange multipliers similarly Degree Preserving Model With and Without The Contact Constraint we arrive at

$$k_{i}^{*}=\sum_{j}p_{ij}=\sum_{j}\frac{e^{-d_{ij}/d_{0}}e^{-\theta_{i}/\theta_{j}}}{1+e^{-d_{ij}/d_{0}}e^{-\theta_{i}/\theta_{j}}}.$$ (7)

To identify the parameters *d*_0_ and *θ_i_* (via *x*_*i*_ ≡ *e^θ_i_^*), we ran the iterative procedure

$$x_{i}^{t+1}=\frac{1}{k_{i}^{*}}\sum_{j}\frac{1}{1/x_{i}^{t}+x_{i}^{t}e^{d/d_{0}}},$$ (8)

for a range of length scales *d*_0_ and found the parameter that preserves the total edge length (error below .01% of the total edge length for all three organisms). The distance dependence is not a soft or hard constraint that is explicitly preserved in this case, but the distance dependence at the optimal *d*_0_ value (purple line in Figure 3) is similar to the one obtained from data (dark blue line in Figure 3), especially for the mouse. This suggests that restricting the total wiring length might be an important mechanism in shaping the structure of the connectome and that *f*(*d*_*ij*_) =*d*_*ij*_ is a meaningful representation of the wiring cost.

Interestingly, the parameter *d*_0_ that matches the total cost of edges is similar in the fly, mouse, and human (*d*_0_ = 9, 9, and 10 soma size units, respectively). That confirms that soma sizes define a spatial scale relevant to synapse formation.

### Network Measures

Here, we discuss the network measures we use to determine how well the models generalize beyond the explicitly built-in features (Dichio & Fallani, 2024). We show the values of these measures for the connectome datasets in Supporting Information Table S4 and compare them to models by plotting the inverse fold change in Supporting Information Figure S1. In this section, instead of the Euclidean distance between the nodes, we use the *geodesic distance δ_ij_*, defined as the length of the paths between nodes *i* and *j* that contain the minimal number of edges. For example, if node *i* is not connected to node *j*, but they share a common neighbor *k, δ_ij_* = 2.

We use the following measures:

- Size of the largest connected component (# nodes in LCC in Supporting Information Table S4)—the largest set of nodes connected by paths.
- Network diameter (Supporting Information Table S4)—the largest shortest distance between the nodes in the network max(*δ_ij_*).
- Average shortest path (average sh. path in Supporting Information Table S4) refers to the average geodesic distance $\frac{1}{N}\sum_{i\neq j}\delta_{ij}$.
- Global efficiency—a quantity related to the harmonic mean of the geodesic distance defined as $E_{g}=\frac{1}{N\left(N-1\right)}\sum_{i\neq j}\frac{1}{\delta_{ij}}$.
- Local efficiency—an average of the global efficiencies of the subgraphs induced by the neighbors of each node, defined as $E_{loc}=\frac{1}{N}\sum_{i}E_{g}\left(G_{i}\right)$.

Additionally, we use the following network measures related to triadic closure that compare the number of triangles *n*_Δ_ to the number of triplets *n*_Λ_:

- Transitivity—the ratio of the number of triangles and the number of triplets for the entire network: $T=\frac{n_{\Delta }}{n_{\Lambda }}$.
- Average clustering coefficient (Supporting Information Table S4)—the average $C=\sum_{i}C_{i}$ of the clustering coefficients *C*_*i*_ of individual nodes. The individual clustering coefficients are defined as $C_{i}=\frac{n_{\Delta_{i}}}{n_{\Lambda_{i}}}$—the ratio of the number of triangles involving node *i* to the number of pairs of its neighbors. If the node has less than two neighbors (*k*_*i*_ < 2), its clustering coefficient is assigned to zero.

The measures we use are standard tools in network neuroscience (Rubinov & Sporns, 2010) and beyond (Latora, Nicosia, & Russo, 2017). Their calculation is implemented in the NetworkX Python package (Hagberg, Swart, & Schult, 2008).

## ACKNOWLEDGMENTS

We thank Helen S. Ansell for providing the physical boundaries for the volumetric mouse and human datasets and for valuable discussions. We also thank Márton Pósfai, Ivan Bonamassa, and Bingjie Hao for useful discussions. We gratefully acknowledge the support of the NSF-Simons National Institute for Theory and Mathematics in Biology via grants NSF DMS-2235451 and Simons Foundation MP-TMPS-00005320.

## SUPPORTING INFORMATION

Supporting information for this article is available at https://doi.org/10.1162/netn_a_00428.

## AUTHOR CONTRIBUTIONS

Anastasiya Salova: Conceptualization; Formal analysis; Investigation; Methodology; Writing – original draft; Writing – review & editing. István A. Kovács: Conceptualization; Funding acquisition; Investigation; Methodology; Supervision; Writing – original draft; Writing – review & editing.

## FUNDING INFORMATION

Anastasiya Salova: NSF DMS, Award ID: DMS-2235451. Simons Foundation (https://dx.doi.org/10.13039/100000893), Award ID: MP-TMPS-00005320.

## DATA AVAILABILITY

A repository containing the preprocessed data, model outputs, and model code is available at https://zenodo.org/records/13376416.

## Supplementary Material



## TECHNICAL TERMS

Neural connectome: Is a network where nodes represent individual neurons and edges correspond to the synaptic connections between pairs of nodes.
Physical networks: Are networks whose nodes and edges are nonoverlapping physical entities embedded in 3D space.
Neural contactome: Is a network where nodes correspond to individual neurons and edges correspond to neurons being in close enough proximity to form synapses.
Maximum entropy ensembles: Network ensembles preserve select properties of the network while keeping the rest of its structure maximally random. The ensemble is specified by a probability *P*(*G*) of the occurrence of network *G*.
Soft constraints: Network constraints that retain the specified network properties (e.g., degree sequence) on average over the network ensemble.
Graphlet (induced subgraph): A small pattern of connectivity within a larger network. To define a graphlet, both the presence and absence of edges must be specified.
Configuration model: Is a maximum entropy model that preserves the degree sequence of the network. Here, we use the “soft” configuration model that preserves the average degree sequence over the network ensemble.
Erdős-Rényi model: Is a network model where each edge has a fixed probability of being present or absent. Here, we use the *G*(*n*, *p*) version where each pair of nodes in a network of size n is assigned an edge with probability p.
