# Supplementary material for: Combined topological and spatial constraints are required to capture the structure of neural connectomes
Source: Netw Neurosci. 2025 Mar 5;9(1):181–206. doi: 10.1162/netn_a_00428 (PMC11949549; doi:10.1162/netn_a_00428)
Supplement: Supplementary file 1 [file netn-9-1-181-s001.pdf]

Supplementary Materials for Combined topological and spatial constraints are required to capture the structure of neural connectomes

Anastasiya Salova[1] and István A. Kovács[1,2,3,4,*]

[1]Department of Physics and Astronomy, Northwestern University, Evanston, IL 60208
[2]NSF-Simons National Institute for Theory and Mathematics in Biology, Chicago, IL 60611
[3]Northwestern Institute on Complex Systems, Northwestern University, Evanston, IL 60208
[4]Department of Engineering Sciences and Applied Mathematics, Northwestern University, Evanston, IL 60208
[*] istvan.kovacs@northwestern.edu

organism	pc_1	pc_2	pc_3	EVR_1	EVR_2	EVR_3	d_{tr}	d	d_1	d_2	d_3	d^c_{tr}	d^c	d^c_1	d^c_2	d^c_3
**fly**	(-0.47,-0.84,-0.28)	(-0.75,0.22,0.62)	(-0.46,-0.50,-0.73)	0.39	0.33	0.28	10	9	17	10	9	12	9	14	10	10
**mouse**	(0.15,0.96,0.23)	(-0.98,0.15,0.04)	(-0.00,-0.23,-0.97)	0.63	0.23	0.14	10	9	15	10	8	12	10	17	10	8
**human**	(0.71,0.25,-0.08)	(-0.71,-0.7,0.08)	(-0.00,-0.11,0.99)	0.71	0.25	0.05	12	15	21	9	2	15	19	26	10	3

Table S1: Spatial orientation of neurons and distance dependence. Here, distance dependence is calculated along the vectors associated with the neuron orientation in space, as defined by the dominant PCA vector of their mesh vertices. Then, PCA was performed on the resulting dominant direction vectors, pc_i correspond to the principal component vectors obtained using this procedure. EVR_i are the corresponding explained variance ratios. d is the characteristic distance estimated from $p(d') \propto e^{-d'/d}$ where d' is the Euclidean distance between the neurons in 3D. d_i are the characteristic distances for the 1D Euclidean distance along the principal component directions.

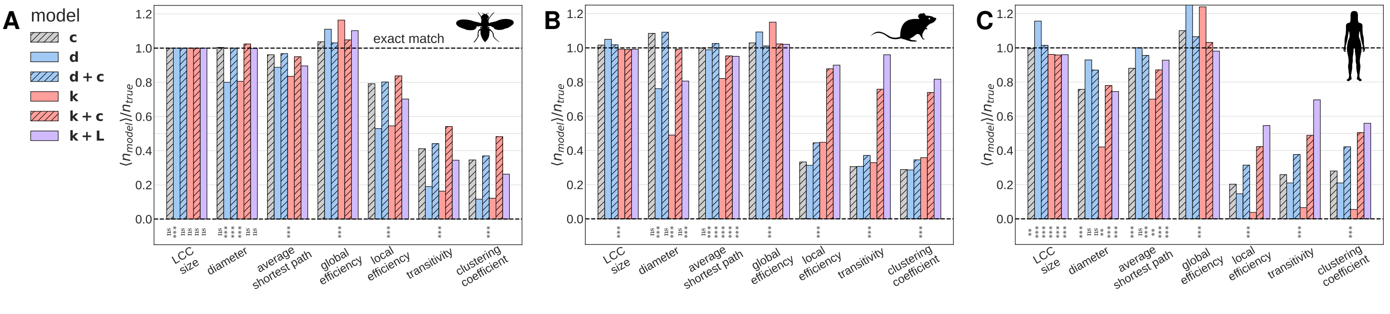

Figure S1: Comparing the basic network properties in empirical connectomes and models. For each model, the property values are averaged over 100 instances, except for the local clustering coefficient of the fly, which was averaged over 5 instances. The definitions of network properties are provided in Network measures.

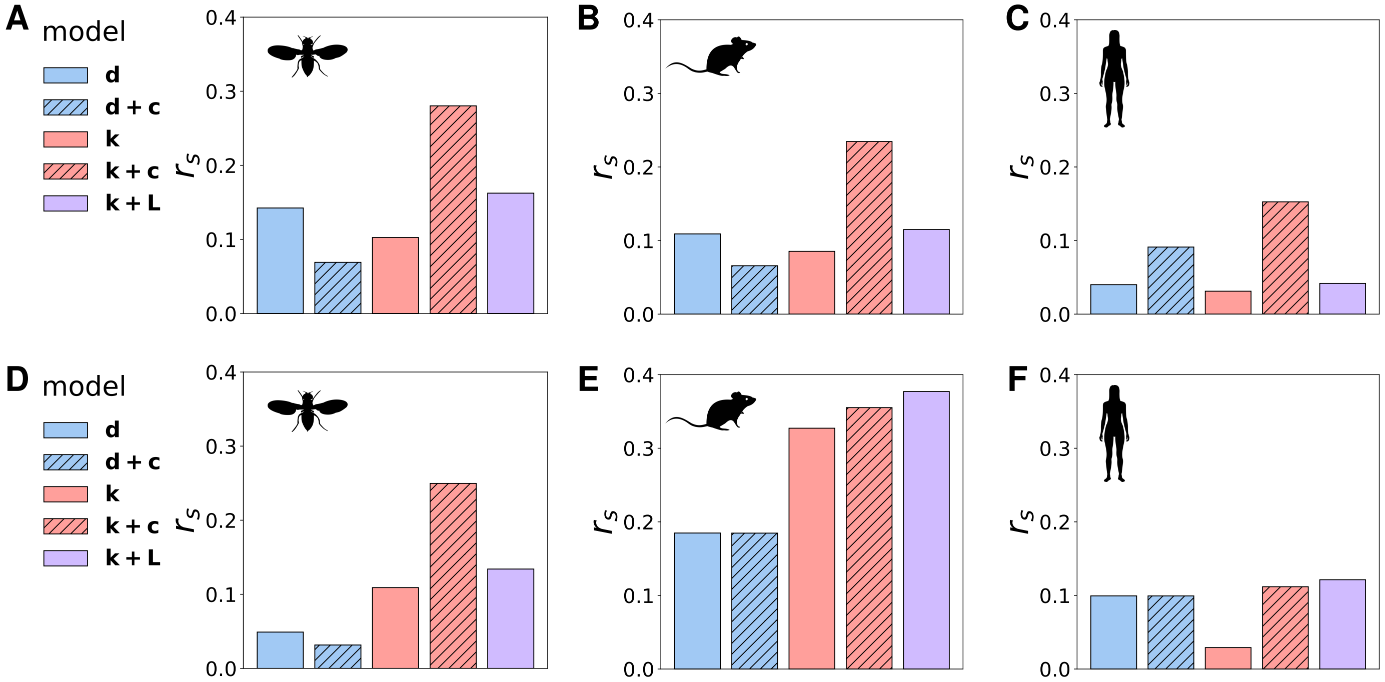

Figure S2: Spearman correlation coefficient between the edge probability predicted by the maximum entropy models and edge weight in data. **A-C**: correlation calculated for all pairs of nodes (models **d**, **k**, and **k+L**) and pairs of nodes that form contactome edges (models **d+c** and **k+c**). **D-F**: correlation calculated for pairs of nodes that form connectome edges.

model	network	AUC-ROC, fly	AUC-ROC, mouse	AUC-ROC, human	AUC-PR, fly	AUC-PR, mouse	AUC-PR, human
**d**	contact	0.86	**0.95**	**0.96**	0.29	**0.62**	0.32
**k**	contact	0.70	0.73	0.79	0.15	0.17	0.07
**k+L**	contact	**0.89**	**0.95**	**0.96**	**0.40**	**0.62**	**0.48**
**d**	synaptic	0.85	0.94	0.97	0.10	0.07	0.03
**k**	synaptic	0.75	0.84	0.86	0.06	0.07	0.01
**k+L**	synaptic	**0.90**	**0.96**	**0.99**	**0.18**	**0.25**	**0.09**
**d+c**	synaptic	0.54	0.57	0.64	0.30	0.10	0.06
**k+c**	synaptic	**0.68**	**0.75**	**0.73**	**0.42**	**0.24**	**0.12**

Table S2: List of AUC-ROC and AUC-PR values for predicting the network edges from models. Rows 1-3: predicting contactome edges from connectome models, see Fig. S7 for the ROC and precision-recall curves. Rows 4-6: predicting connectome edges from connectome models, see Fig. S6. Rows 7,8: predicting connectome edges from connectome models restricted by the contactome, see Fig. S10. The values corresponding to the model with the best prediction within each category are shown in bold font for each organism.

model	fly	mouse	human
**d**	-8,350,235	-498,369	-429,298
**k**	-9,286,707	-579,990	-548,372
**k+L**	**-7,441,092**	**-418,911**	**-364,679**
**c**	-4,356,676	-387,194	-321,470
**d+c**	-4,323,885	-384,247	-312,048
**k+c**	**-4,055,652**	**-342,900**	**-292,252**

Table S3: Log-likelihood values of the empirical connectome for different models. The values corresponding to the model with the highest log-likelihood within each category are shown in bold font for each organism.

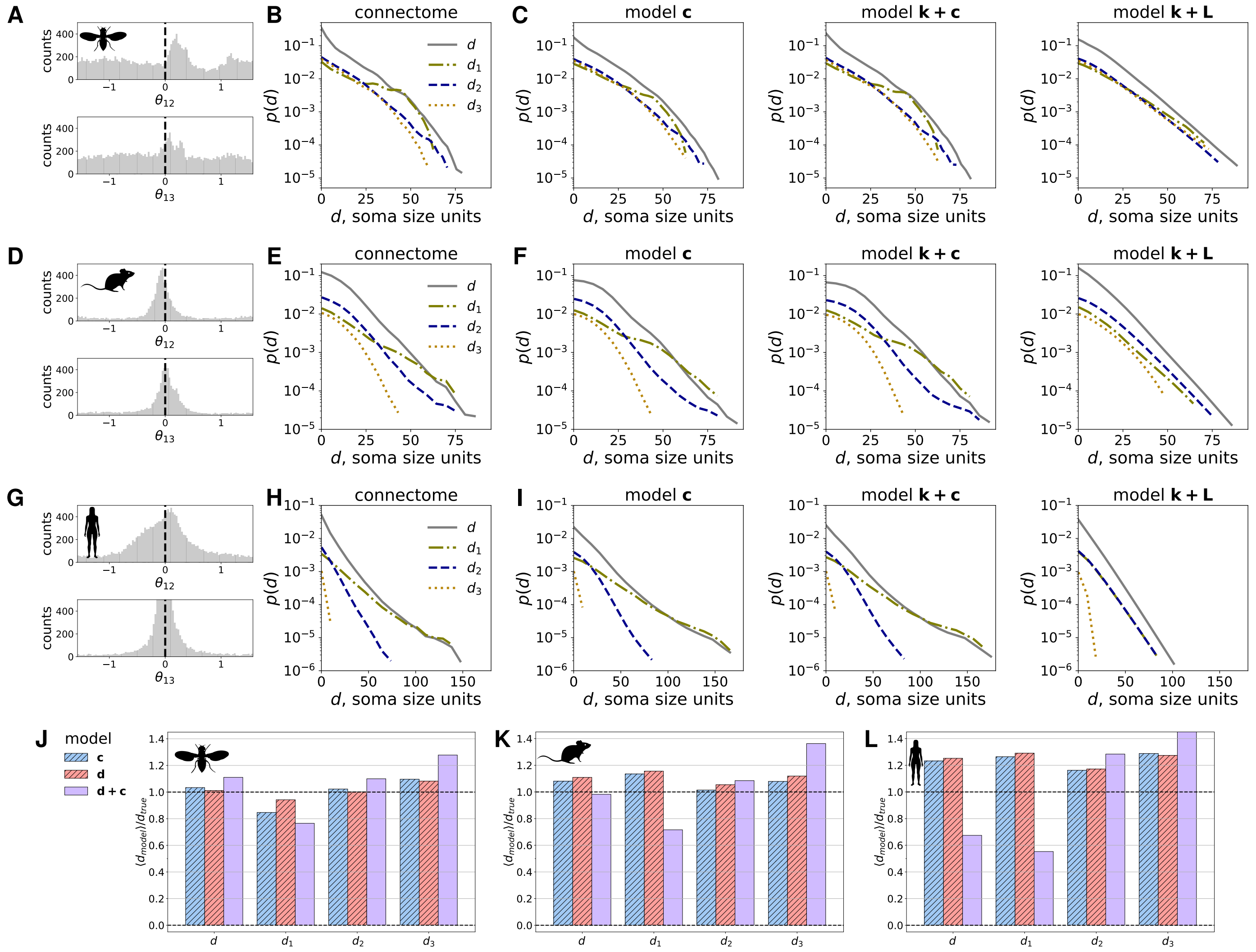

Figure S3: Heterogeneity and neuron orientation dependence in distance dependence for fly (first row), mouse (second row) and human (third row). **A, D, G**: distribution of angles θ_{1i} between the dominant PCA vectors of individual neuron meshes and pc_1, the dominant principal component obtained from calculating the PCA of all the individual neuron orientations in the pc_1-pc_2 plane (top) and pc_1-pc_3 plane (bottom). See Table S1 for the PCA vector values and more details in the caption. **B, E, H**: 3D Euclidean distance dependence (grey) and the distance dependence in pc_1, pc_2, and pc_3 directions (olive, dark blue, and dark yellow) in the connectome. **C, F, I**: distance dependence for models **c**, **k+c**, and **k+L**. **J-L**: inverse fold changes for the characteristic distances in 3D and along the principal components. See Table S1 for the d_i values.

organism	#nodes in LCC	diam.	average sh. path	global efficiency	local efficiency	transi-tivity	clust. coeff.	# triangles	# squares, no diagonal	# squares, one diagonal	# squares, two diagonals
**fly**	16,804	5	2.53	0.42	0.64	0.21	0.30	52,367,260	2,616,817,833	5,405,355,056	781,627,819
**mouse**	6,224	12	3.32	0.30	0.26	0.10	0.11	366,256	8,804,181	14,863,110	863,837
**human**	13,352	21	5.60	0.14	0.06	0.05	0.04	24,242	253,578	71,290	2,012

Table S4: Properties of the synaptic and contact networks used in Figures 4 and S1. For definitions of network metrics used in this table, see Network measures.

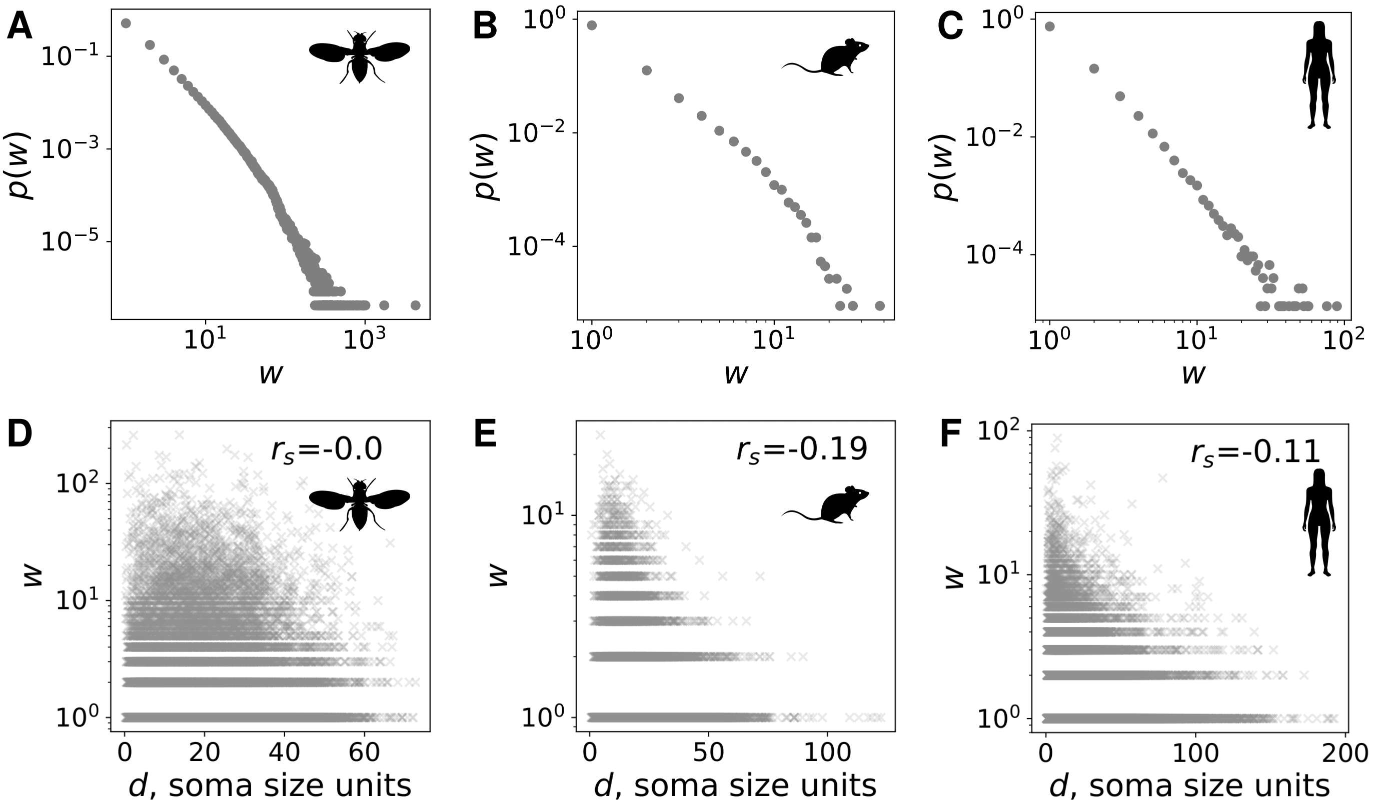

Figure S4: Degree distribution and distance dependence for edge weights. Here, edge weight is defined as the number of synapses between a given pair of neurons. Synapse strength can also be quantified by its size in mammalian brains, thus other definitions of edge weight could also be appropriate. **A-C**: distribution of edge weights. **D-F**: edge weight as a function of distance.

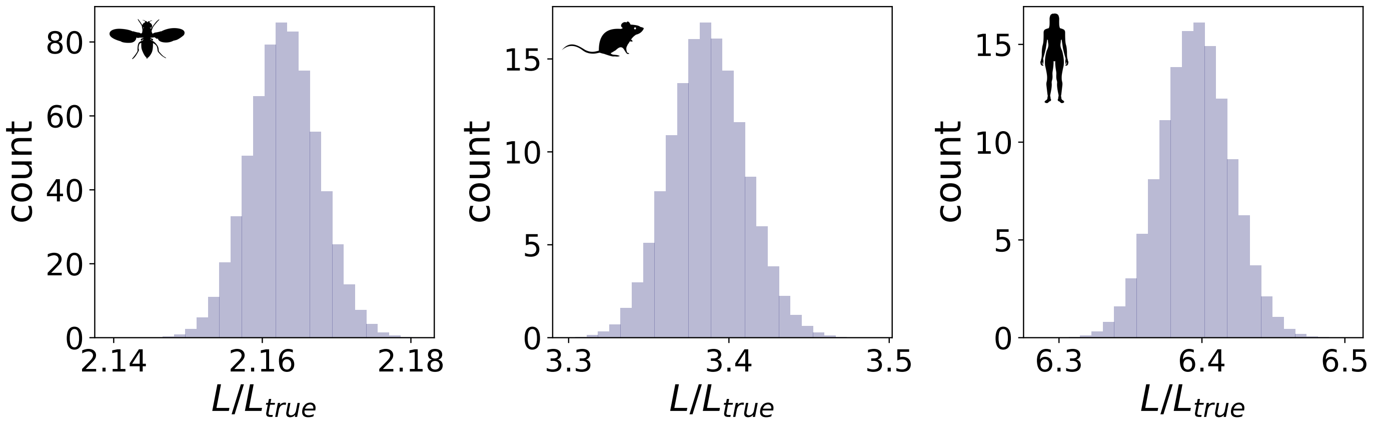

Figure S5: Distribution of total wiring lengths obtained by randomly shuffling the neuron positions while fully preserving the network topology. 200,000 randomized spatial networks were obtained for each organism.

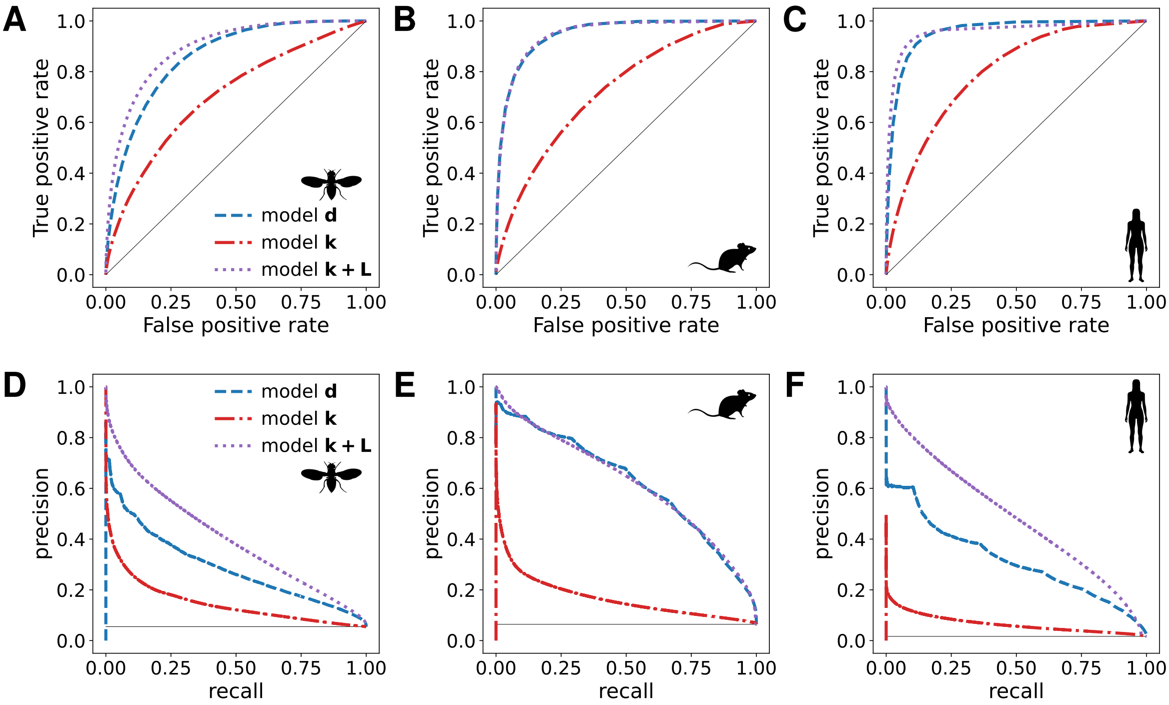

Figure S6: Predicting connectome edges from connectome models. **A-C:** ROC (receiver operating characteristic) curves for model **d** (blue), model **k** (red) and model **k+L** (purple). **D-F:** precision-recall curves for the same models. The areas under the curve (AUC) for the three models are provided in Table S2

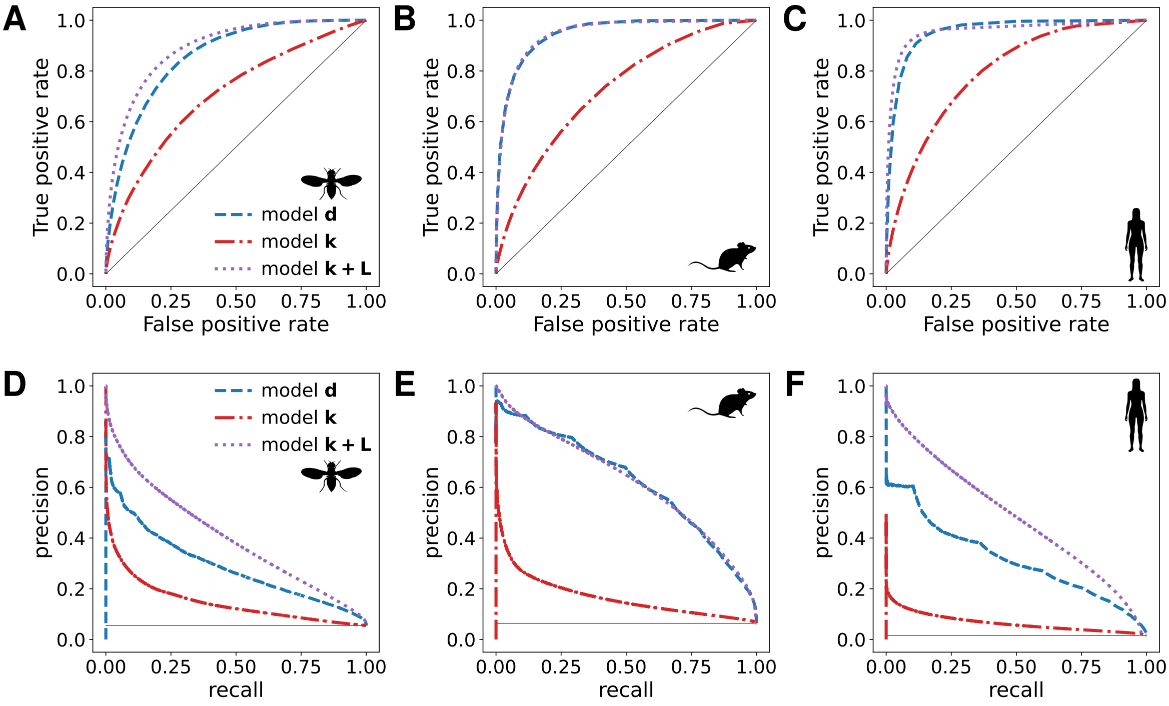

Figure S7: Predicting contactome edges from connectome models. **A-C:** ROC (receiver operating characteristic) curves for model **d** (blue), model **k** (red) and model **k+L** (purple). **D-F:** precision-recall curves for the same models. The areas under the curve (AUC) for the three models are provided in Table S2

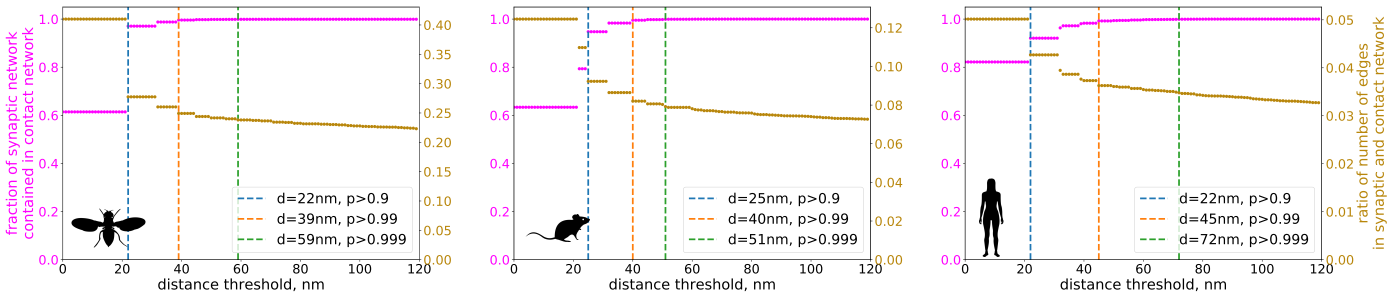

Figure S8: Thresholding the contact network. We demonstrate the fraction of the synaptic network that is contained in the contact network for different thresholds of distances corresponding to a contactome edge (magenta). We also show the ratios of the number of edges in the synaptic network to that of the contact network at different thresholds (dark yellow). Finally, we provide the distance values at which 90, 99, and 99.9% of the synaptic network is contained in the contactome (vertical lines). To construct our final contact network, we find the union of the synaptic network and the contact network thresholded at the distance corresponding to 99% of the synaptic edges contained in the contact network.

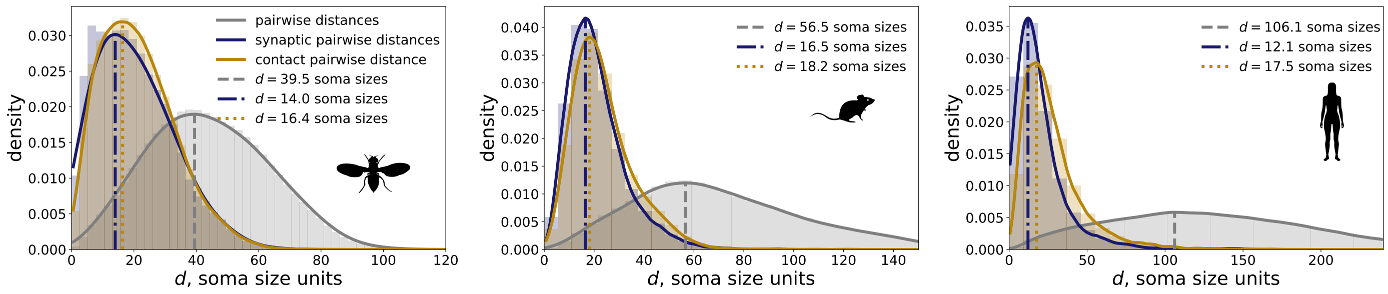

Figure S9: Distribution of distances between all pairs of neurons (grey), pairs of neurons connected by connectome edges (dark blue), and pairs of neurons connected by contactome edges (dark yellow). The locations of the peaks of these distributions were estimated using kernel density estimation. The peaks of the synaptic and contact pairwise distances represent the typical distances between the neurons in the connectome and contactome. The peak of the entire distance distribution (grey) represents the distance above which the effects of the finite size of the experimental volume affect the inferred distance dependence.

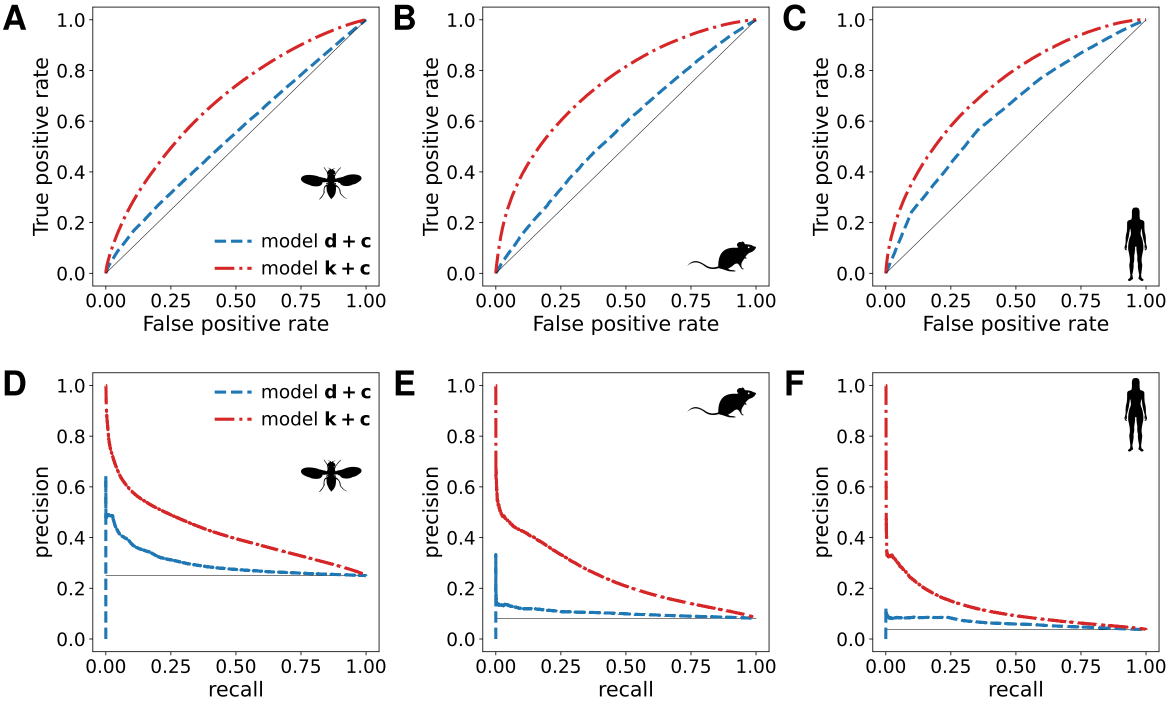

Figure S10: Predicting connectome edges from connectome models with contactome constraints. **A-C:** ROC (receiver operating characteristic) curves for model **d+c** (blue), model **k+c** (red). **D-F:** precision-recall curves for the same models. The areas under the curve (AUC) for the three models are provided in Table S2

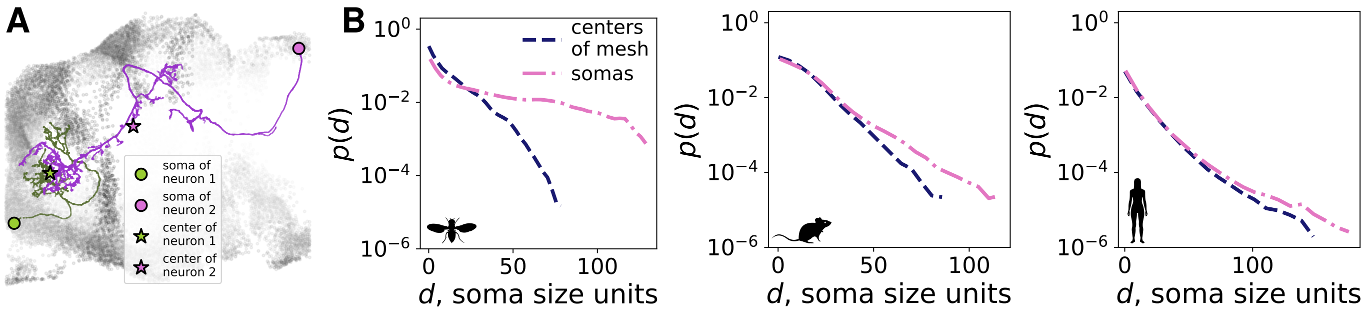

Figure S11: Distance dependence for different definitions of neuron locations. **Left**: an example of two neurons (neuron 1 in green, neuron 2 in purple) in the fly brain. The distance between their centers of mesh (shown as stars) is significantly smaller than the distance between their somas (circles). The positions of somas of all the uncropped neurons are shown in light grey. **Right**: distance dependence using soma locations (purple) vs centers of mesh (dark blue).

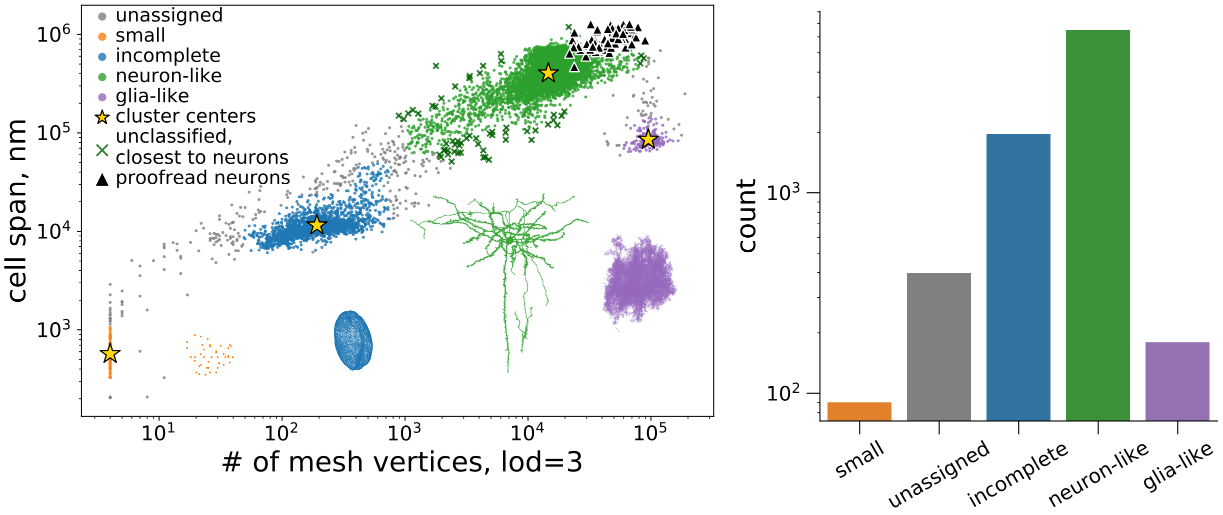

Figure S12: Classification of mouse segments labeled as "neurons". Left: distinct clusters of cells (shown in different colors) as identified by DBSCAN based on the"cell span" (cell size in the leading PCA vector direction, where the PCA is performed on the mesh vertices for lod=3) and the number of mesh vertices for the same resolution, together with examples of each type of cell shown in the same color as their clusters. Right: number of cells of each type.

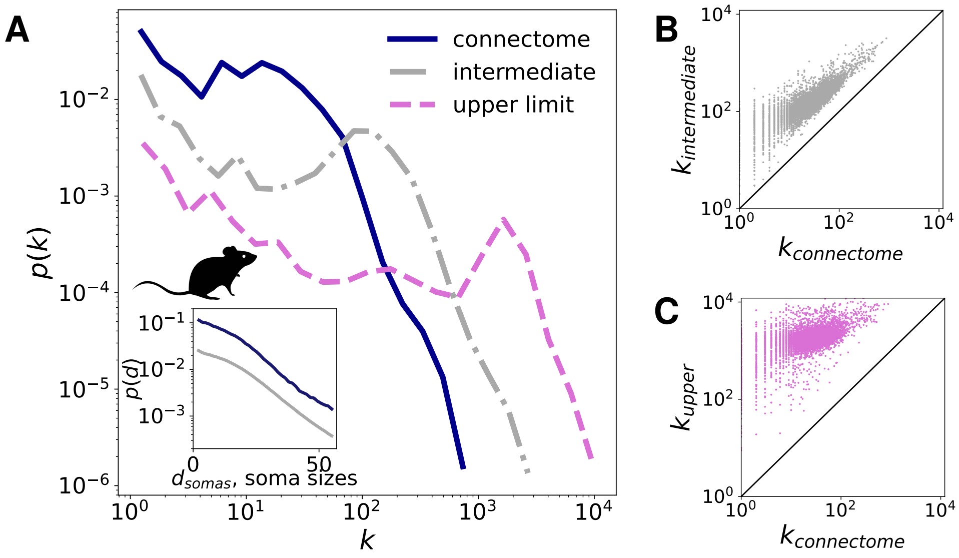

Figure S13: Comparing the node degrees in the mouse connectome we use and its upper bounds. In all cases, the degree distribution is shown for the uncropped neurons as defined in Synaptic network construction: topology. In the degree calculation, the connectome degree distribution (dark blue) accounts for the neighbors that are themselves uncropped neurons. The "intermediate" degree distribution (grey) includes all neighbors with a single soma in the experimental volume. The "upper limit" degree distribution (pink) accounts for all neighbors assigned a unique id. **A**: degree distributions. Inset: distance dependence for the connectome and the intermediate network. Here, distance is defined as the Euclidean distance between somas. The estimated exponential scales d_0 ($p(d) \propto e^{-d/d_0}$) are similar—11 soma sizes for the connectome and 12 soma sizes for the intermediate network. **B**: degree correlation between the connectome and intermediate network. Pearson correlation coefficient is $r_p = 0.89$. **C**: degree correlation between the connectome and upper limit network, $r_p = 0.55$.

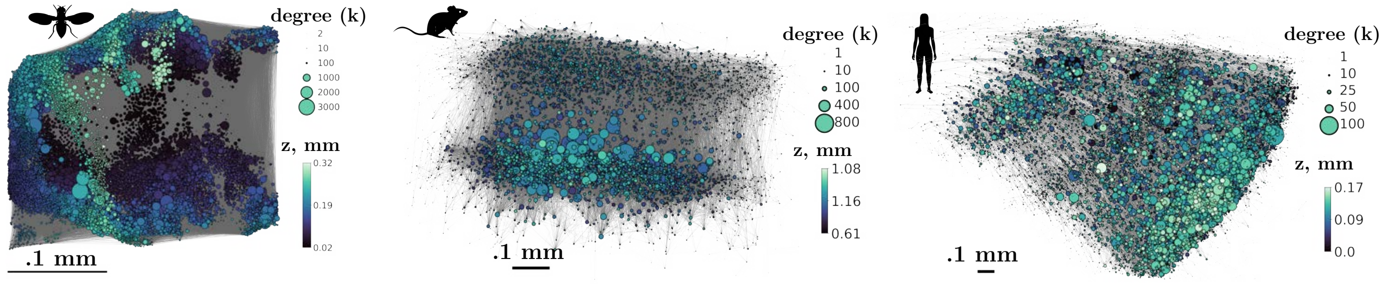

Figure S14: Synaptic networks from soma positions. Here, node positions correspond to the xy location of neuron somas, node color and size correspond to their undirected degree and z position respectively.

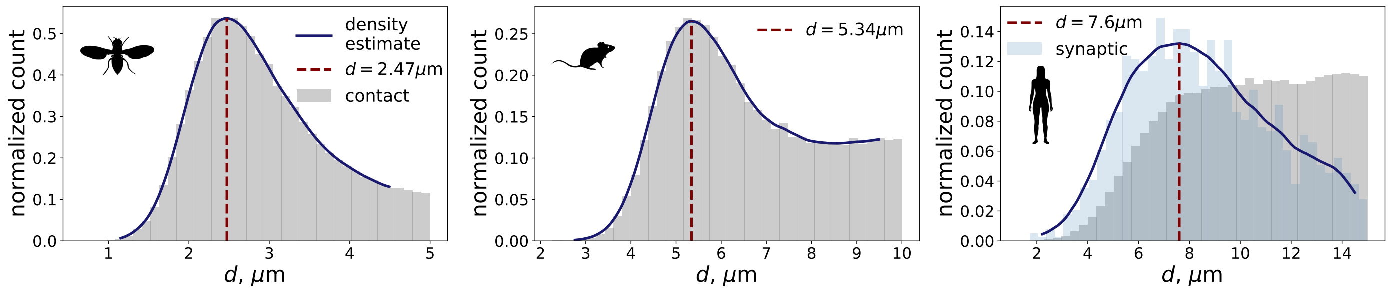

Figure S15: Soma size estimation. Grey: binned distances from contact to soma (only plotted for visualization purposes). Blue: kernel density estimation using linear kernel forms. Red: location of the peak of the density function that we use as a soma size. d stands for the distance from contact to the center of the soma.

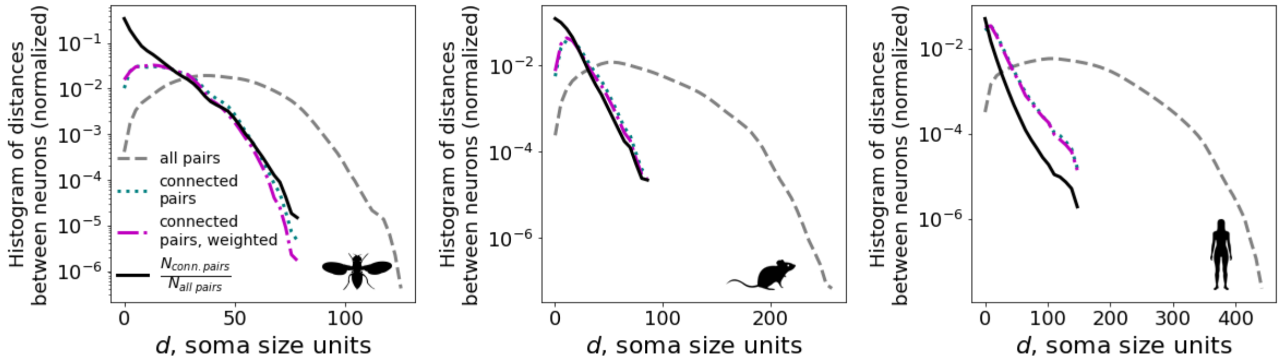

Figure S16: Distance distribution histograms. Dashed grey: all pairwise Euclidean distances between neuron centers of mesh. Teal: distribution of pairwise Euclidean distances between connected pairs. Magenta: distribution of pairwise Euclidean distances between connected pairs weighted by the number of synapses between each pair. Black: probability of forming at least one edge as a function of distance (our $p(d)$).
